# Taxonomic and functional structure of macrobenthic invertebrate communities and their response to environmental variables along the subbranches of the Nile River (rayahs), Egypt

**DOI:** 10.1007/s11356-022-24140-z

**Published:** 2022-11-19

**Authors:** Reda E. Bendary, Shaimaa M. Ibrahim, Mohamed E. Goher, Hosam E. Elsaied, Gamal M. El Shabrawy, Mohamed Abd El Mordy, Magdy T. Khalil

**Affiliations:** 1 Hydrobiology Lab., Freshwater & Lakes Division, National Institute of Oceanography and Fisheries (NIOF), Cairo, Egypt; 2 Chemistry Lab., Freshwater & Lakes Division, National Institute of Oceanography and Fisheries (NIOF), Cairo, Egypt; 3 Genetics and Genetic Engineering Lab., Aquaculture Division, National Institute of Oceanography and Fisheries (NIOF), Cairo, Egypt; 4 Department of Zoology, Faculty of Science, Ain Shams University, Cairo, Egypt

**Keywords:** Biological trait analysis, Functional diversity, Macroinvertebrates, Environmental variables, Rayahs canals

## Abstract

Macrobenthic invertebrate communities serve as markers of anthropogenic stress in freshwater ecosystems. In this study, 17 sampling sites were selected from two Nile river subbranches (El-Rayah El-Behery and El-Rayah El-Nassery) and subjected to different anthropogenic influences to explore the ecological environment and characteristics of macrobenthos communities. Macrobenthos were studied using taxonomic diversity and biological trait analysis to investigate how human activity and variation in water quality affect their structure and function. A total of 37 taxa represented by 43,389 individuals were recognized. The communities are composed chiefly of Oligochaeta and aquatic insects. Multivariate statistical analyses found that the most influential environmental variables in the structural and functional community were sodium, dissolved oxygen, silicate, pH, calcium, and cadmium. At high levels of pollution, notably sewage and industrial pollution in the northern part of El-Rayah El-Behery, characteristics such as larger body size, detritus feeders, burrowers, and high tolerance to pollution predominated, whereas at low levels of pollution, features such as small body sizes, scraper and predator feeders, intolerant and fairly tolerant of pollution, and climber and swimmer mobility are predominant. The results confirm our prediction that the distribution of macroinvertebrate traits varies spatially in response to environmental changes. The diversity-based method distinguished impacted sewage and industrial sites from thermal effluent sites, while the trait-based approach illustrated an apparent variance between the ecological status of contaminated regions. Therefore, the biological features should be employed in addition to structural aspects for assessing the biodiversity of macroinvertebrate communities under environmental stressors.

**Supplementary Information:**

The online version contains supplementary material available at 10.1007/s11356-022-24140-z.

## Introduction

Macrobenthic invertebrates are considered efficient markers to assess and screen the health of freshwater ecosystems because they are prevalent and sensitive to environmental stressors (Piló et al. 2016; Borja et al. 2010). These motivate significant river ecosystem cycle processes, such as organic material retention and breakdown, nutrient and mineral recycling, and contribution to energy processing at various trophic levels (Butkas et al. 2011; Mangadze et al. 2019). Recent studies illustrated a considerable correlation between these invertebrates and ecological issues. Therefore, this correlation analysis has significantly improved river ecosystems (Butkas et al. 2011). Assessing the functional features and structure of the macrobenthic assemblages can highlight how ecological changes affect benthic assemblages (Piló et al. 2016; Kenny et al. 2018; Hu et al. 2019). Several authors concluded that the macrobenthic functional traits could illustrate the environmental disturbance better than these macrobenthic invertebrates themselves (Piló et al. 2016; Nasi et al. 2018; D’Alessandro et al. 2020). There is a strong relationship between macrobenthic invertebrates’ functional features, environmental processes, and ecosystem functions (Cadotte et al. 2011). This macrobenthos can respond swiftly to many stressors (Mouillot et al. 2013; Voß and Schäfer 2017).

Most ecological research focuses on structural diversity, abundance, and biomass of species communities, whereas functional adaptation to environmental variables is rarely evaluated. The functional structure of the macrobenthos is expressed via a collection of traits that define the morphological and behavioral features of the taxa. The stability and function of communities and ecosystems are influenced by their characteristics and interactions (Loreau et al. 2001), which also demonstrate a correlation between alterations in the macrobenthic community and environmental factors (Lavorel and Garnier 2002). Biological trait analysis (BTA) is a representative trait-based technique that utilizes collections of the biological traits of organisms to illustrate the variation pattern of the functional features along spatial or temporal gradients (Bremner et al. 2006). BTA and taxonomic analysis should be integrated (Díaz and Cabido 2001; Villéger et al. 2010). Functional data is quantitatively characterized as a set of biological features, with values supplied for each taxon based on lifestyle and activity mode. Integrating the functional characteristics of species composition with all species’ biomass or abundance values allows for their quantification. This expression, like structural data, can be used to study gradients and connections to environmental variables. Consequently, the methodology establishes links among species, environmental conditions, and ecological activities. However, BTA was initially developed and used for freshwater systems (e.g., Zhang et al. 2021; Laini et al. 2019; Lamouroux et al. 2004; Charvet et al. 2000; Usseglio-Polatera et al. 2000), but BTA in the macrobenthic community does not perform on River Nile before.

The Nile River is the principal supply of fresh water for all purposes and uses in Egypt, supplying the country with more than 95% of its freshwater needs. Many branches, irrigation canals, and streams originate from the Nile to form the agricultural system, extending for 31,000 km (Goher et al. 2019). The irrigation canals and rayahs, like the Nile River, are primarily used for drinking, irrigation, industry, navigation, and fishing. Consequently, due to the increased population and industrial development, massive organic and inorganic waste and heavy metals are thrown into the Nile and travel into the Mediterranean Sea via Sudan and Egypt (Goher et al. 2019, 2021). Metals are more concentrated in sediments than in the water body in rivers and watercourses (Shyleshchandran et al. 2018). They are discharged into the water body under appropriate hydrological and chemical circumstances, resulting in water pollution and harming aquatic organisms (Kouidri et al. 2016). Nutrient, metals, and organic pollution can cause variations in macrobenthos community structures by decreasing the abundance of sensitive species, species richness, and diversity while increasing the abundance of tolerant species (Birk et al. 2020). The authors hypothesized that the diversity-based and trait-based methods would describe the response of the macrobenthic community to different environmental stresses in various approaches.

Therefore, in this study, the diversity-based and trait-based methods were performed (1) to assess the variations in taxonomic and functional macroinvertebrate community structures toward different stressors within El-Behery and El-Nassery rayahs, (2) to determine the significant environmental factors prompting macrobenthos species and their biological traits, and (3) identify the most sensitive traits to environmental stressors.

## Materials and methods

### Study area and sampling locations

**Fig. 1:**
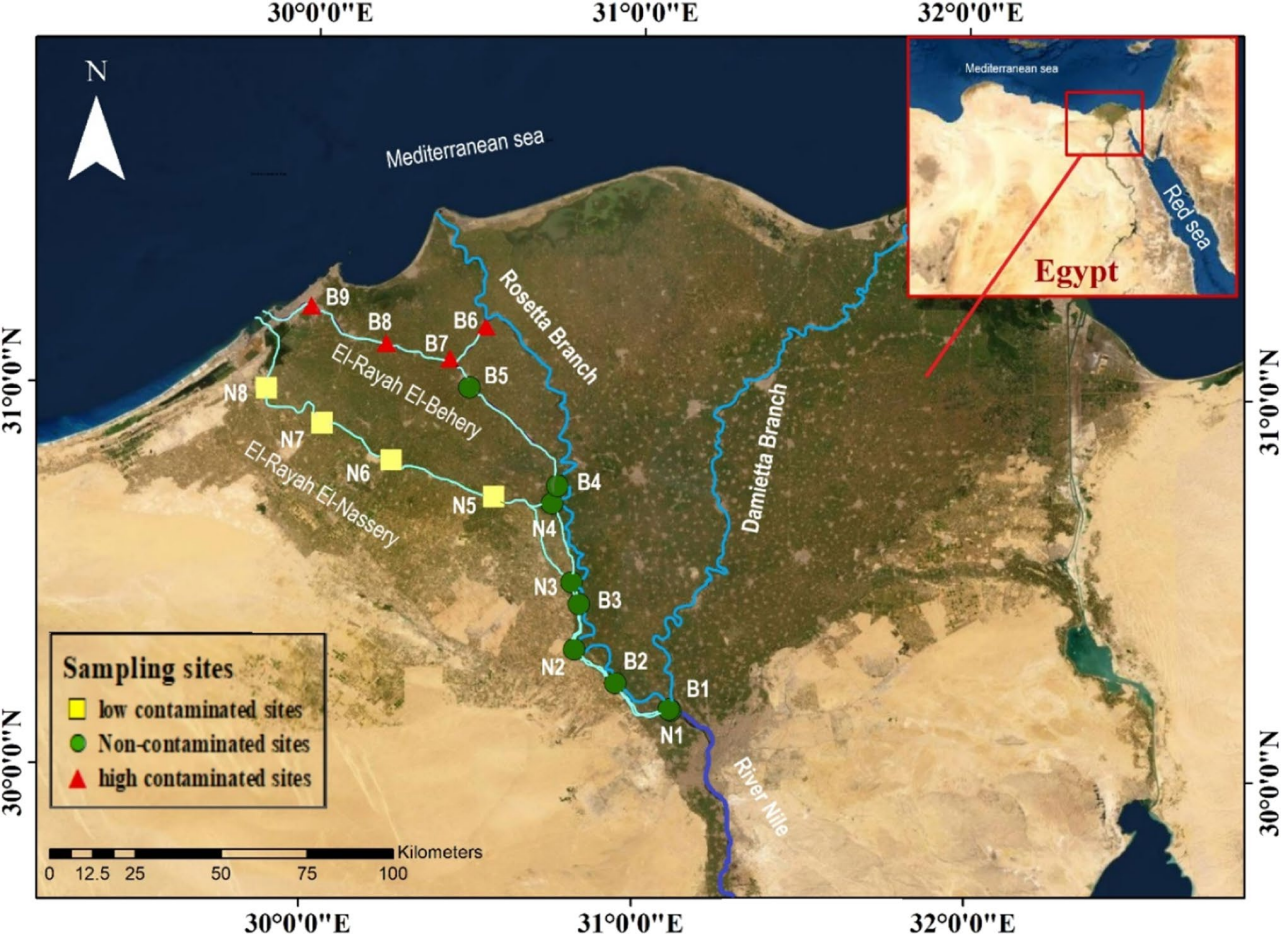
Map of the study area indicating the sampling sites in El-Rayah El-Behery (B1-B9) and El-Rayah El-Nassery, (N1-N8), Nile River

In the Delta region, the Nile is divided into two main branches and four subbranches (irrigation canals, or rayahs in the colloquial dialect): the El-Behery, El-Nassery, El-Tawfiky, and El-Menoufy rayahs (Talab et al. 2016; Bendary and Ibrahim 2021). The El-Behery and El-Nassery rayahs provide fresh water for millions of people in the West Delta area and the Alexandria Governorate (Goher 2015). Both rayahs arise from the Rosetta branch downstream from the Delta Barrage, heading northwest to the Mediterranean Sea. El-Rayah El-Behery is 215 km long that is refilled with water from the Rosetta branch at Damanhur Governorate via the El-Mahmoudia Canal. The El-Mahmoudia Canal is the northern portion of El-Rayah El-Behery (from sites B6–B9). It originated from the Rosetta branch and is considered a multi-stressor area in our study due to the discharged sewage and industrial wastes via Rosetta branch. Unfortunately, Rosetta branch collects massive amounts of pollutants and wastewater from several sources, including the El-Rahawy Drain (El Sayed et al. 2020). El-Rayah El-Nassery is 200 km long and connects to the El-Nubaria Canal, which splits off from El-Rayah El-Behery at Bolin Bridge and runs to the Mediterranean Sea via Mariout Lake. The section from sites N4 to N8 is called El-Nubaria Canal. The EI-Nubaria canal is vital for fishing and agriculture; however, it receives different wastewater from the surrounding farmlands, electric power plants, and small water treatment stations (see Fig. 1 and Table 1 for more details).

**Table 1:** Characteristics of the 17 sampling sites included in the present study

Site	Name	Latitude	Longitude	Distance (km)*	Description
B1	El Kanater	30° 10′ 47.36ʺ	31° 6′ 18.69ʺ	3–4	It runs between agricultural and industrial regions/receives urban and industrial effluent
B2	Abo Ghaleb	30° 14′ 46.90ʺ	30° 56′ 33.68ʺ	30	Downstream Abu-Ghalib electrical plant/agricultural region on the east side
B3	Kafr Dawood	30° 27′ 3.75ʺ	30° 49′ 41.35ʺ	60	It flows between agricultural and residential regions
B4	El-Tawfikia	30° 48′ 36.91ʺ	30° 45′ 21.65ʺ	100	At El-Tawfikia bridge/beside agricultural way (Cairo-Alexandria)
B5	Damanhur 1	31° 00′ 46.5ʺ	30° 28′ 52.8ʺ	135	At the entrance of Damanhour city
B6	El-Mahmoudia	31° 10′ 25.9ʺ	30° 31′ 42.1ʺ	145	1 km downstream the originate of El-Mahmoudia Canal from the Rosetta branch
B7	Damanhur 2	31° 5′ 16.85ʺ	30°25′16.79ʺ	140	Downstream the confluence of RB with El-Mahamoudia/Middle of a crowded residential region
B8	Kafr El-Dawar	31° 7′ 31.58ʺ	30° 13′ 31.82ʺ	170	Between agricultural and residential regions at the two sides/low water level
B9	El-Siuof (Alexandria)	31° 13′ 6.67ʺ	29° 59′ 39.74ʺ	205	Upstream the end of El-Rayah/Next to many water and electricity plants/low water level
N1	El Kanater	30° 10′ 36.78ʺ	31° 6′ 29.77ʺ	2	Between residential regions
N2	El-Khatatba	30° 19′ 57.76ʺ	30° 48′ 57.5ʺ	40	Between agricultural and residential regions
N3	Koum Hamada	30° 30′ 31.3ʺ	30° 48′ 18.6ʺ 70	70	Located before the confluence of El-Rayah with El-Nubaria Canal/surrounded by agricultural regions
N4	El-Noubaria 1	30° 42′ 51.0ʺ	30° 44′ 26.8ʺ	93	The beginning of the El-Nubaria Canal/swift water flow
N5	El-Noubaria 2	30° 43′ 44.69ʺ	30° 33′ 47.14ʺ	90	It receives wastes from Kom Hamada electricity plant/downstream meeting of El-Rayah with El-Nubaria Canal
N6	Itai El-Baroud	30° 49′ 4.75ʺ	30° 14′ 57.56ʺ	115	Between agricultural and residential regions
N7	HoushIssa	30° 54′ 38.06ʺ	30° 2′ 9.52ʺ	140	Between agricultural and residential regions
N8	The end of the Nubaria canal	30° 59′ 54.76ʺ	29° 51′ 49.97ʺ	190	Before El-Nubaria’s current lock (El-Haouies)

^*^Distance downstream of the Delta barrage

According to the contamination degree (*C*_d_) and potential ecological risk (RI) indices (El Sayed et al. 2022) and water quality (WQ) Egyptian standards (Habash et al. 2018), the study area sites are classified into three main sectors: sector 1 includes sites from B6 to B9 (El-Mahmoudia Canal) with the highest values of contaminations; sector 2 includes sites from N5 to N8 (from El-Nubaria Canal) which recorded the lowest values of contaminations, and sector 3 contains the remains that considered as non-contaminated sites.

### Sampling

Nine sites from El-Rayah El-Behery (B1–B9) and eight sites from El-Rayah El-Nassery (N1–N8) were sampled. Sampling was performed seasonally in the spring, summer, and autumn of 2014 and winter of 2015. Water samples were collected using an A 2 L polyvinyl chloride water sampler. An Ekman Grab sampler collected samples of macrobenthic invertebrates (225 cm^2^). Each specimen was sieved through a 0.5-mm screen and stored in a 10% buffered formalin. In the laboratory, macroinvertebrate individuals were counted and classified to the species or genus level as far as possible.

### Environmental data

The following variables were measured as discussed in American Public Health Association–defined methods (APHA 2005): water temperature (°C), pH, total dissolved solids (TDS), dissolved oxygen (DO), chemical oxygen demand (COD), ammonia (NH_4_–N), nitrogen dioxide (NO_2_–N), phosphate (PO_4_–P), silicate (SiO_4_), sulfate (SO_3_), calcium (Ca), chlorine (Cl), sodium (Na), potassium(K), lead (Pb), and cadmium (Cd) as shown in the supplementary information (ST1), to inspect the effect of anthropogenic stressors on the macrobenthic species’ distribution and their biological traits.

### Biological traits analysis (BTA)

Ten biological traits were chosen depending on their ability to define the critical biological and environmental processes related to anthropogenic and ecological environmental stresses: morphology, body size, longevity, feeding mode, mobility, habitat, substrate affinity, reproductive technique, larval environmental development, and pollution tolerance. The 10 traits were divided into 40 categories that represented the behavior and strategy of the organisms in greater detail. For example, the “feeding mode” trait categories were filter feeders, detritus feeders, collectors/gatherers, scrapers/grazers, engulfers/predators, and scavengers (Table 2). The association of each taxon with the categories of each trait was quantitively scored based on the “fuzzy coding” method (Chevenet et al. 1994; Bremner et al. 2003). Each feature was allocated a functional biological score ranging from 0 to 3, where 0 displays no affinity, 1 or 2 represents moderate affinity, and 3 illustrates a high relationship for the specified trait category. For the BTA, three matrices were created: the density of taxa in each station (matrix “taxa by stations”), the functional biological score of each taxon (matrix “taxa by traits”), and a functional traits score combined with the abundance value for all constituent species in each station (matrix “traits by stations”) (Bremner et al. 2003). To equilibrium the abundance values between abundant and rare species without losing density effects, the initial matrices were transformed by log (1 + *x*) before the calculations. These three matrices were created for four seasons to determine seasonal impacts. Biological trait data from a range of published resources, journal articles, and online databases (https://www.epa.gov/risk/freshwater-biological-traits-database-traits) were collected to generate the “taxa by traits” matrix.

**Table 2:** A list of Biological traits and categories used in the macrobenthic community of El-Rayah El-Behery and El-Rayah El-Nassery

Traits	No	Category	Trait code
Morphology	1	Soft	M1
	2	Exoskeleton	M2
Body size(mm)	1	< 5	S1
	2	5–10	S2
	3	10–20	S3
	4	> 20	S4
Longevity (year)	1	< 1	L1
	2	1–2	L2
	3	3–10	L3
	4	> 10	L4
Feeding mode	1	Filter-feeder	F1
	2	Detritus feeder	F2
	3	Collector/gatherer	F3
	4	Scraper/grazer	F4
	5	Engulfer/predator	F5
	6	Scavenger	F6
Mobility	1	Burrower	mob1
	2	Clinger	mob2
	3	Scrawler	mob3
	4	Crawler	mob4
	5	Climber	mob5
	6	Swimmer	mob6
Habitat	1	Burrow-dwelling	H1
	2	Free living	H2
	3	Epiphytic	H3
	4	Attached to substratum	H4
Substrate affinity	1	Coarse, clean sand	SA1
	2	Fine, clean sand	SA2
	3	Sandy mud	SA3
	4	Muddy sand	SA4
	5	Mud	SA5
Reproductive technique	1	Sexual (Gonochoric)	R1
	2	Sexual: Hermaphrodite	R2
Larval development	1	None (brooding)	LD1
	2	Bentonic	LD2
	3	Planktonic	LD3
Pollution tolerance	1	Intolerant of pollution (0–2)	P1
	2	Moderately intolerant (3–5)	P2
	3	Fairly tolerant (6–8)	P3
	4	Very tolerant (8–10)	P4

### Statistical analysis

Fuzzy correspondence analysis (FCA) was utilized to arrange the “traits by stations” matrix on a two-dimensional plane. Which traits influenced the species’ distribution inside and between the various sites in rayahs and seasons are clear. The FCA’s correlation ratio (CR) represents each trait’s contribution to the total variance. The features with a CR value larger than 0.1 are regarded as having the most influence on the variation among sites. FCA was conducted using R-4.1.2 (ADE4-ADE4TKGUI) (Thioulouse et al. 1997).

Multivariate statistical analyses were conducted using Canoco 4.5 software (Ter Braak and Smilauer 2002). Principal component analysis (PCA) was used to evaluate seasonal and spatial patterns of environmental factors. Redundancy analysis (RDAs) was done to highlight the correlations between macrobenthic species and environmental factors, likewise the correlation between trait features and environmental factors. RDAs was suitable for data analysis as in the preliminary detrended correspondence analysis (DCA) for species composition and trait; the lengths of the gradient for the first axis was less than 3 SD (Lepš and Šmilauer 2003). Data for the PCA and RDAs were log (*x* + 1) transformed. The significance of the variables was determined using a Monte Carlo permutation test with 499 permutations, and only the significant environmental factors were plotted in the RDAs.

Non-parametric Kruskal–Wallis tests were done to show the significant differences between the sampling sites and seasons for the environmental variables and macrobenthos structural and trait composition. Statistical calculations were performed using SPSS 24. All statistical analysis was done at a 95% confidence interval.

## Results

### Environmental variables

**Fig. 2:**
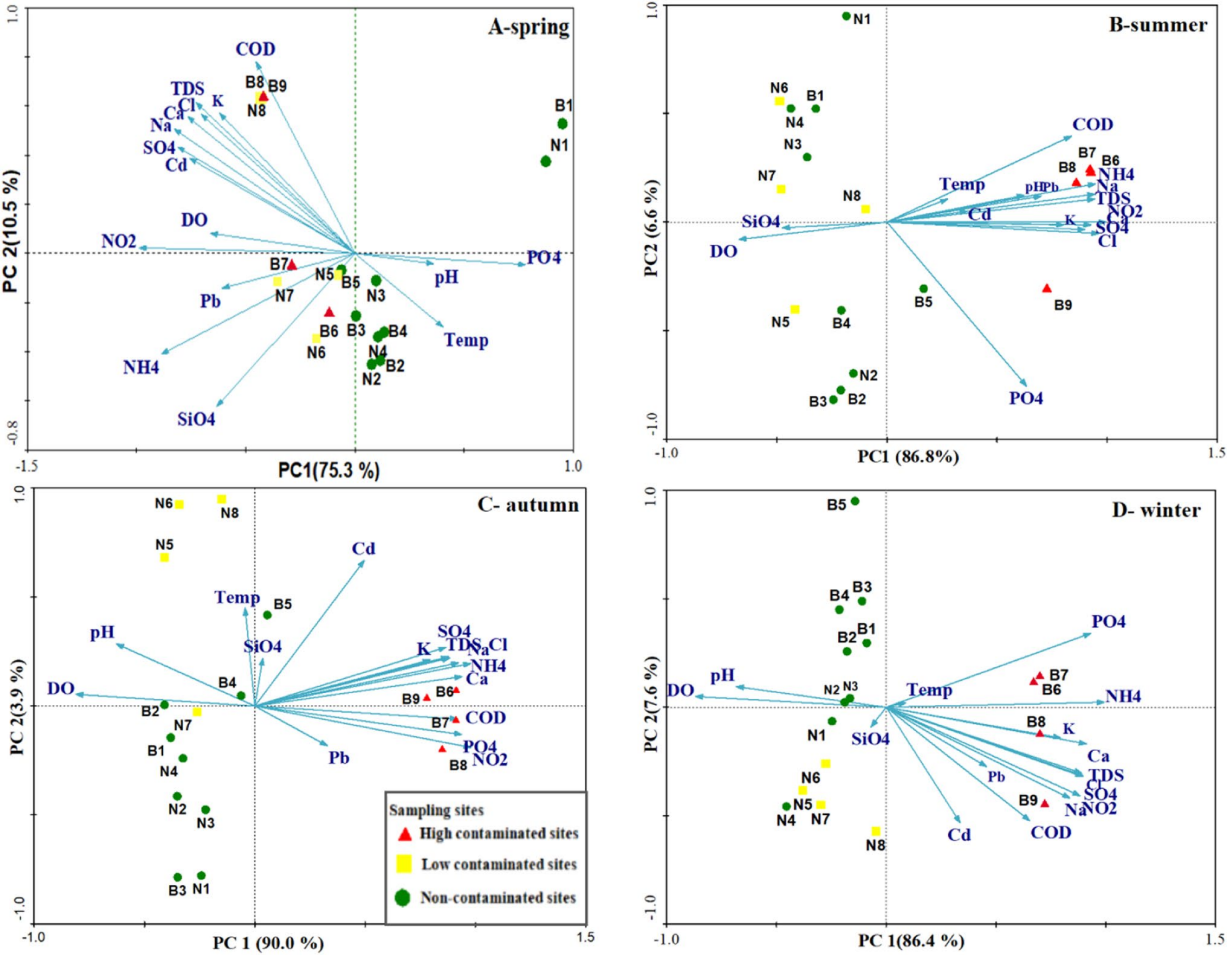
Principal component analysis plots of the relevant environmental variables in the El-Rayah El-Behery and El-Rayah El-Nassery for different seasons

Among environmental factors, remarkable variations were found across sampling sites and seasons (Supplementary Tables S1–S5). Figure 2 illustrates the PCA plots of environmental data for the rayahs canals in different seasons. The first two axes of the PCA explained 85.8 to 93.9% of the overall variability. El-Mahmoudia Canal sites B6, B8, and B7 were associated with higher average values of NH_4_ (2827 µg/l), NO_2_ (166 µg/l), and PO_4_ (89 µg/l), respectively, than other sites (*p* < 0.05). Likewise, B9 showed a lower average content of DO (3.2 mg/l) (*p* < 0.05) and the highest average value of Ca (40.4 mg/l) (*p* < 0.05). El-Nubaria Canal sites N8 and N4 had higher average value of TDS (506 mg/l) and SiO_4_ (3.5 mg/l), respectively (*p* < 0.05). All environmental variables differed significantly between seasons (*p* < 0.05) except cadmium. The maximum values of NH_4_ (6733 mg/l), TDS (637 mg/l), and Ca (47.8 mg/l) were highest in the winter, while the values of NO_2_ (447 mg/l), Pb (60 µg/l), and pH (8.8) were highest in the autumn, and the highest value of SiO_4_ (5.4 µg/l) occurred in summer.

### Taxonomic composition and seasonal variations of the macrobenthic community

**Fig. 3:**
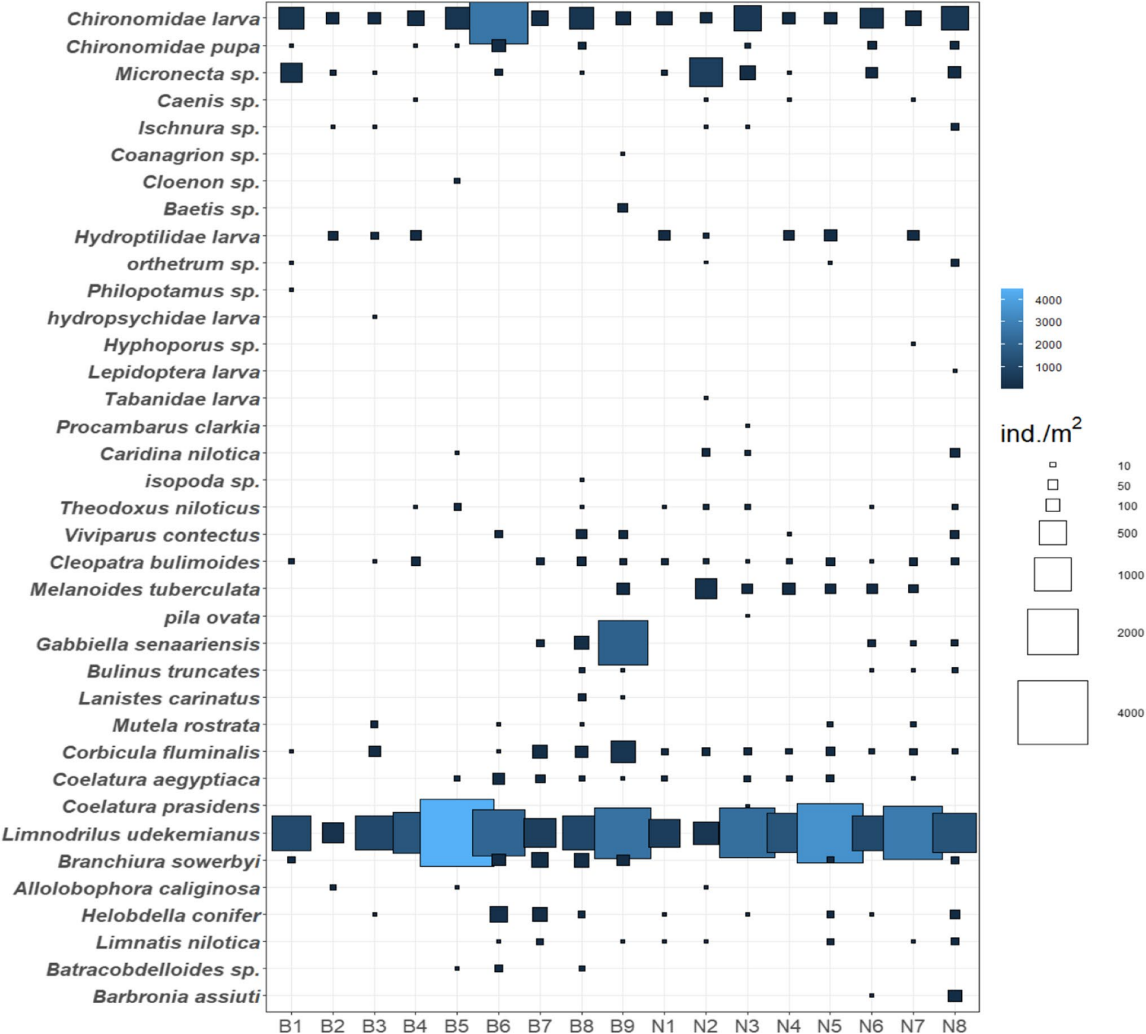
Taxa distribution within El-Rayah El-Behery and El-Rayah El-Nassery stations. The size of the squares is proportional to the density (the bigger squares, the higher density)

An overall of 37 taxa was recorded (43,389 individuals/m^2^) belonging to the Insecta, Crustacea, Gastropoda, Bivalvia, and Oligochaeta (Suppl., Table S5). Oligochaeta generally dominated (seven species, 1772 individuals/m^2^, 69%), especially *Limnodrilus udekemianus*. The second-largest group was Insecta (15 species, 506 individuals/m^2^, 20%), with the dominance of Chironomidae larvae. Gastropoda is represented by eight species (201 individuals/m^2^, 8%), and Bivalvia is represented by four species (67 individuals/m^2^, 3%). Crustacea (three species, 6 individuals/m^2^) were the least abundant group. A significant difference among the sampling sites was noticed in the species composition of macrobenthos (*p* < 0.05). The highest density was recorded at site B9 (5281 individuals/m^2^), followed by B6 (5262 individuals/m^2^). Site N8 had the most remarkable diversity (18 species). Site B2 contained the lowest number of taxa and density (six species and 476 individuals/m^2^). The highly tolerant species, such as *L. udekemianus* and Chironomidae larvae, were the taxa with the maximum frequency, disseminated at all the sites. The sensitive polluted species such as *Cloenon* sp., *Caenis* sp., and Hydropsychidae larvae rarely occurred and disappeared in El-Mahmoudia Canal sites. The species abundance and diversity over the two rayahs increased from the southern sites toward the northern sites, which were mainly characterized by oligochaetes species (Fig. 3). A significant difference between El-Mahmoudia Canal sites and the other remaining sires was observed (*p* < 0.05). The distribution of benthic macrofauna in the irrigation rayahs varied greatly between seasons. Commonly, the abundance and highest number of taxa were recorded in spring and winter related to summer and autumn (Suppl. Table S6). The highest diversity and density were observed during the winter (3254 individuals/m^2^ and 29 species). The lowest density was noted during the autumn (1921 individuals/m^2^), while the lowest number of taxa was observed during the spring (21 species). *L. udekemianus* and Chironomidae larvae were the dominant species in all seasons. The most abundant individuals in the winter were *Micronecta* sp., averaging 313.2 individuals/m^2^. The most abundant individuals in the summer were *G. senaariensis*, averaging 473.5 individuals/m^2^.

### Fuzzy correspondence analysis (FCA)

**Fig. 4:**
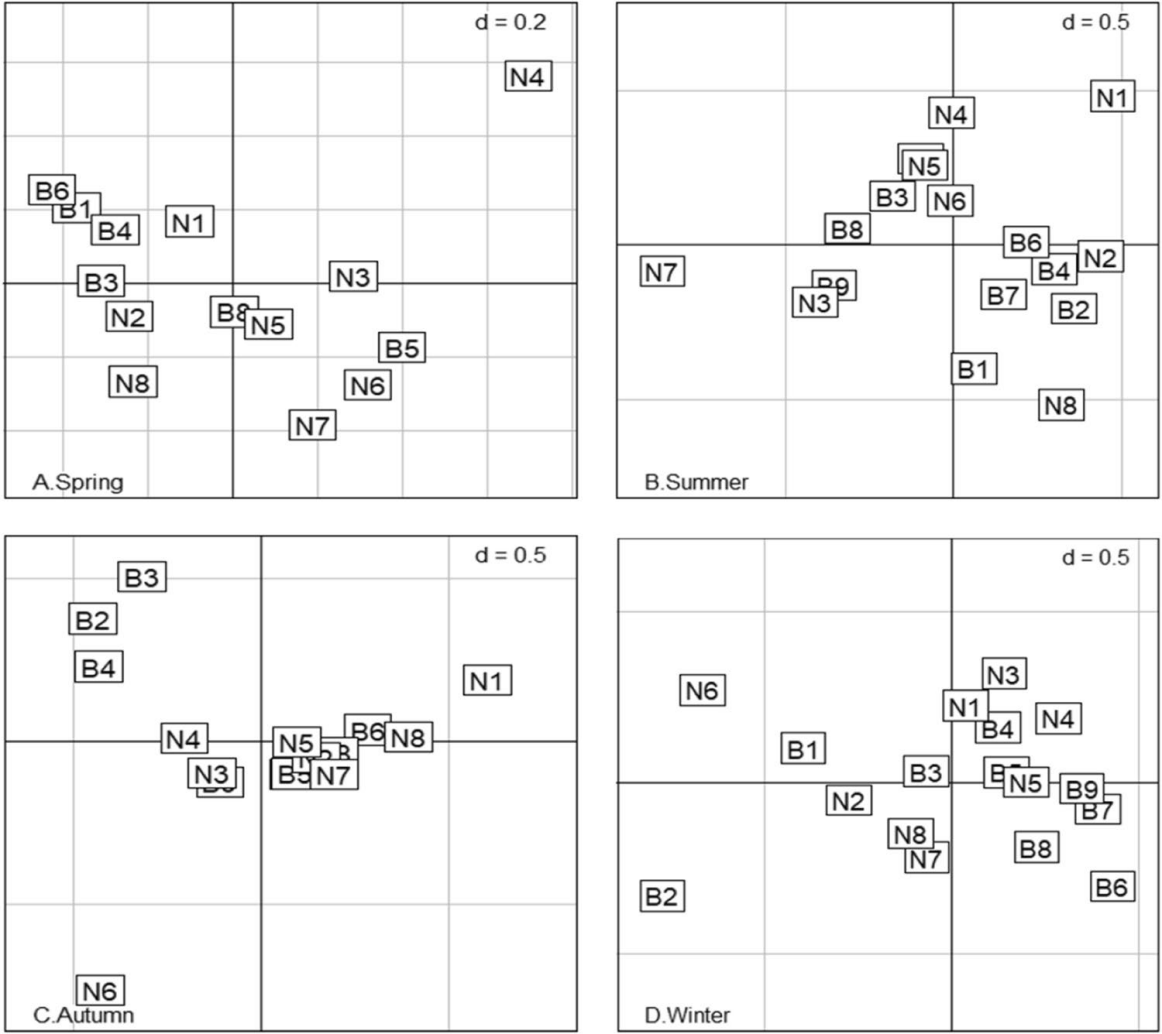
Factor map of the sampling stations obtained by FCA for different seasons: **A** spring, **B** summer, **C** autumn, and **D** winter

Biological trait scores obtained using the “fuzzy coding” technique are shown in the supplementary data (Table S7). In all seasons, the chi-square test (or Fisher’s exact test) was done for all traits and revealed a significant correlation (*p* < 0.001) between trait categories and sample sites. FCA for the matrix (“traits by stations”) was conducted separately for the four seasons (Fig. 4). In the spring, the first two FCA axes expressed 59% of the overall variance, with 39% accounted for by axis 1 and 20% by axis 2. The influence of each trait on this variance is explained in the CR (Table 3). The main biological features separated along FCA axis 1 were body size, mobility, longevity, and substrate affinity. In the summer, the FCA axes represent 68% of the overall variance; axis 1 counted 53% and axis 2 15% of the variability. The biological traits on FCA axis 1 were morphology, body size, habitat, reproductive technique, and larval development. Mobility was highly correlated with both axes. In the autumn, 55% of the overall variance was expressed by the first two FCA axes: 36% by axis 1 and 19% by axis 2. Body size, longevity, and mobility were correlated with axis 1, and habitat and larval development were associated with axis 2. Larval development was connected with axes 1 and 2, and pollution tolerance and substrate affinity were not correlated with axes 1 and 2. During the winter, the first two FCA axes represented 64% of the overall variance. Axis 1 explained 45% of the overall variability in body size, longevity, mobility, habitat, reproductive technique, and larval development. Body size distribution and longevity categories along axis 2 explained 19% of the overall variability.

**Table 3:** Correlation ratios (CR) of traits on the first two FCA axes and the biological traits for all seasons

	Spring		Summer		Autumn		Winter	
Correlation ratios	Axis I	Axis 2	Axis I	Axis 2	Axis I	Axis 2	Axis I	Axis 2
Relative inertia (%)	39	20	53	15	36	19	45	19
Morphology	0.0042	0.0469	**0.1575**	0.0276	0.0145	0.0380	0.1015	0.0012
Body size (mm)	**0.2121**	**0.0959**	**0.1973**	0.0707	**0.1506**	0.0447	**0.2093**	0.0845
Longevity (year)	**0.1761**	**0.0998**	0.0474	0.0587	**0.1767**	0.0822	**0.2429**	0.0807
Feeding mode	0.0810	0.0184	0.1061	0.0565	0.0359	0.0114	0.0363	0.0235
Mobility	**0.2690**	0.0590	**0.2290**	**0.1351**	**0.1510**	0.0890	**0.1165**	0.0600
Habitat	0.0154	0.0324	**0.1383**	0.0787	0.0962	**0.1156**	**0.1376**	0.0075
Substrate affinity	**0.1105**	0.0144	0.0294	0.0065	0.0444	0.0030	0.0282	0.0026
Reproductive technique	0.0011	0.0650	**0.1157**	0.0525	0.0756	0.0538	**0.1397**	0.0025
Larval development	0.0160	0.0529	**0.1465**	0.1029	0.0985	**0.1904**	**0.1421**	0.0397
Pollution tolerance	0.0417	0.0270	0.0133	0.0996	0.0143	0.0360	0.0132	0.0596

Bold figures indicate CR > 0.1

The high-contaminated sector had similar distributions of traits, dominated by species tolerant of pollution, detritus feeders, burrowers, mud substrate affinity, and large body size (P4, F2, mob1, SA5, and S4). At the same time, the low-contaminated sector is characterized by the following trait categories, very tolerant and fairly tolerant of pollution, filter-feeder, scraper/grazer, and climber (P4, P3, F2, F4, and mob5). The non-contaminated sites had higher percentages of small body size, engulfer/predator and scavenger feeders, and swimmers (S1, F5, F6, and mob6) than the El-Mahmoudia canal and El-Nubaria Canal sites. A significant difference among seasons was observed for the trait categories of macrobenthos (*p* < 0.05). Regarding the different contaminated sectors, Kruskal–Wallis test exposed that there were significant variances (*p* < 0.05) in trait categories (i.e., S3, L2, L4, F1, F4, H2, H4, SA2, mob4, and LD3).

### Associations between species and environmental variable

**Fig. 5:**
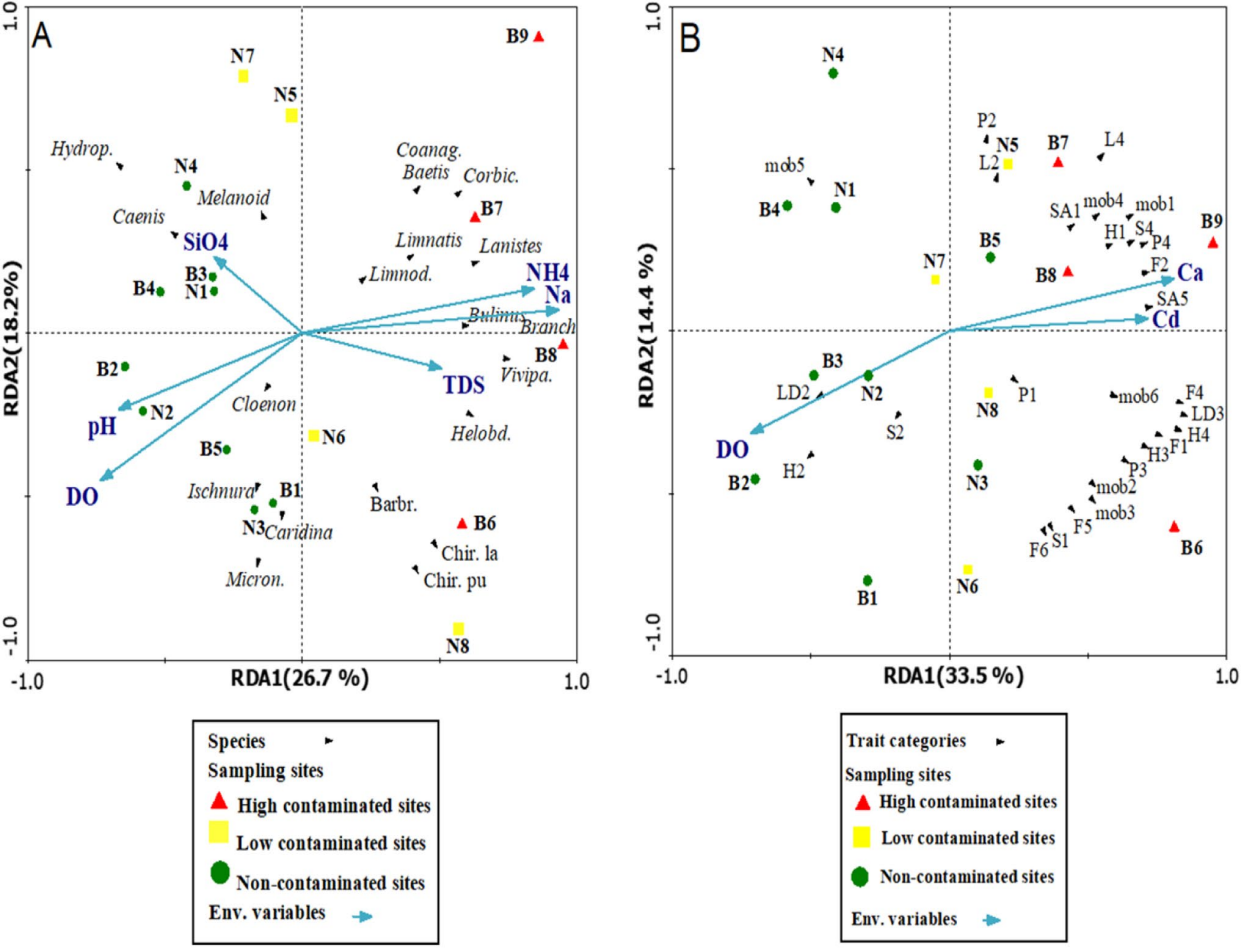
**A** RDA triplot illustrating associations between macroinvertebrate species and the most significant environmental variables. See Fig. 3 for complete species names. **B** RDA triplot explaining associations between macroinvertebrate traits and the most important environmental variables. See Table 2 for the meaning of the trait codes

RDA was conducted to study the correlations between macrobenthic invertebrate species and environmental factors (Fig. 5A). The cumulative variance of taxa data along the first two axes and the cumulative variance of the taxa-environment relationship along the first two axes constituted the same percentage, 44.9% of the total variability. The arrows describe the environmental factors and point in the direction of the factor with the maximum variation. Na, DO, SiO_4_, and pH were the greatest significant variables in taxa occurrence (*p* < 0.05). Monte Carlo permutation tests demonstrated that Na was the most significant environmental factor associated with community composition (*p* < 0.05; *F*-ratio = 4.64). The second most significant factor (*p* < 0.05; *F*-ratio = 1.99) was DO, which influenced the overall variance of species composition. The plot shows that strong relationships existed between (NH_4_, Na, and TDS) and the individuals of (*Branchiura sowerbyi, Bulinus truncates, Viviparus contectus*, *Lanistes carinatus*, and *Helobdella conifer*). The DO was the most effective variable for the distribution of *Micronecta* sp., *Cloenon* sp., *Ischnura* sp., and *Caridina nilotica*. SiO_4_ was the most effective variable for distributing *Hydroptilidae* larvae, *Caenis* sp., and *Melanoides tuberculata*.

### Relations between traits and environmental variable

Based on RDA analysis, the cumulative percentage variance along the first two axes recorded 47.9% of the total variance of macrobenthic trait categories, and the cumulative percentage variance of trait features-environment relation along the first axes 1 and 2 recorded 59.2% of the total variance. The RDA results showed that Ca and Cd (*p* < 0.05; *F*-ratio = 4.9 and 2.02, respectively) significantly affected the trait categories distribution. Burrow-dwelling, mud substratum affinity, burrowers, detritus feeder, > 20-mm-large body size, and very tolerant pollution (H1, SA5, mob1, F2, S4, and P4) were associated with high levels of Ca and Cd. Free living, bentonic larval development, and small body size (5–10 mm) (H2, LD2, S2) were related to the DO concentration (Fig. 5B).

## Discussion

### Environmental variables of El-Rayah El-Behery and El-Rayah El-Nassery

El-Mahmoudia Canal was the most polluted area in our study. High concentrations of nitrogen and phosphorous nutrient salts besides cations and anions caused due to these numerous anthropogenic stressors (Goher et al. 2021), affecting biodiversity and causing disruption in macroinvertebrates (El-Shabrawy and Goher 2012; El-Otify and Iskaros 2015). El Sayed et al. (2022) also recorded that the various anthropogenic stressors in El-Mahmoudia Canal contributed to higher aggregations of heavy metals. The highest concentrations of most abiotic parameters were recorded in the El-Mahmoudia Canal, indicating its state of constant contamination. This result is in line with El Sayed et al. (2020) in Rosetta branch and (Talab et al. 2016) in the rayahs. The low DO levels in this canal may be correlated with the extreme transmission of organic pollutants and nutrients. In this study, the highest average value of silica was recorded at site N4 (3.5 mg/l), and the lowest average value was noted at site B5 (1.7 mg/l) in contravention with other nutrients; this indicated that sewage and industrial effluents do not significantly affect the distribution of reactive silica in irrigation canals, where the accurate concentrations of river silica levels were shown to be dependent on the motion of underlying soils and the degradation of silicate rocks (Wang et al. 2013; El-Otify and Iskaros 2015).

### Macrobenthic invertebrate community structures

The quantitative and qualitative investigation of the macrobenthos assemblage in El-Behery and El-Nassery rayahs demonstrated that it was mainly composed of Oligochaeta and aquatic insects. *L. udekemianus* and Chironomidae larvae were the most abundant and widely distributed species. The community composition of macrobenthos in the irrigation canals of the Nile River has not changed much since previous investigations (Mola and Abdel Gawad 2014; Saad Abd El-Halim et al. 2015; Khalifa and Bendary 2016; El-Damhogy et al. 2017; Bendary and Ibrahim 2021). Yap et al. (2006) described that *Limnodrilus* sp. are contaminant-resistant species that can survive in habitats with poor water quality. Rashid and Pandit (2014) reported that oligochaetes were the most common benthic macroinvertebrates because they can live in many different environments and tolerate hypoxia due to the breakdown of organic matter in the background. Al-Shami et al. (2010) reported high populations of *Chironomus* sp. in six Malaysian rivers as the water quality changed from high to moderate contamination. Chironomidae are poor water quality indicators in freshwater environments because of their abundance in sewage-polluted areas (Serra et al. 2017). This dominance of Chironomidae and Oligochaeta was also recorded in other rivers affected by anthropogenic stressors, e.g., the Langat River, Malaysia (Azrina et al. 2006), the Bílina River, Czech Republic (Orendt et al. 2012), the Bzura River, Poland (Grzybkowska et al. 2015), and the Hex River, South Africa (Erasmus et al. 2021).

The reduction in species diversity and abundance at site B2 could be due to the thermal effluence from the electricity plant. Worthington et al. (2015) and Han et al. (2017) stated that the thermal discharge reduced the abundance and richness of macrobenthic invertebrates. Increased Ca levels can induce shell calcification in warm waters (Abd El-Wakeil et al. 2015), which could explain the rise in molluscan quantity at thermal-polluted site B9. Hussein et al. (2011) stated that freshwater snails display a high degree of tolerance and flexibility in a specific range of physicochemical variation. The increase in the quantity of sensitive polluted species Cloenon sp., Caenis sp., and Hydropsychidae larva in the non-contaminated area is explained by Ephemeroptera and Trichoptera taxa increase with an increase in water quality (Erasmus et al. 2021).

### Functional diversity pattern of macrobenthic invertebrates

The current investigation of macrobenthic fauna discovered functional diversity patterns associated with contamination aspects and environmental variables. The maximum characteristic patterns were revealed for body size, mobility, feeding mode, substrate affinity, and pollution tolerance. This outcome is in line with other studies in which traits associated with mobility and feeding have been determined to be the most accountable for discriminating between species communities (Paganelli et al. 2012). In all seasons, however, feeding mode traits were weakly associated with both FCA axes; this may be due to the consistent domination of only categories, e.g., in all samples, taxa with detritus feeders predominated over taxa with other feeding modes. The variety of larvae produced by an organism indicates its response to environmental variations. Taxa with planktonic larval development possess a higher spreading potential and a lower hazard of extinction than organisms with other forms of development (McHugh and Fong 2002); this explains the domination of taxa with planktonic larvae in the sites of high environmental stress in the study area.

BTA showed that trait distributions in the El-Mahmoudia Canal were relatively similar. Eutrophication has been a major environmental problem in this region because of excessive nutrient effluents. Waste effluents carrying inorganic and organic contaminants have been shown to impact macrobenthic species assemblages in previous investigations (El Sayed et al. 2020; Bendary and Ibrahim 2021), where the biological traits that reflected the nutrient effluents were mud substratum affinity, large body size, detritus feeders, burrowers, and high tolerance of pollution. The current study’s outcomes align with those of Llanos et al. (2020). Body size is macrobenthos’s most direct and instinctive feature (Edegbene et al. 2020; Odume 2020). Typically, the body size of macrobenthic organisms is correlated with stressor fluctuations, but our results recorded that the body size increased with increasing inorganic and organic contamination. This result is consistent with earlier research indicating that macrobenthic organisms with large body sizes are further common in habitats with somewhat extreme environmental stresses than organisms with small body sizes (Gusmao et al. 2016; Odume 2020; Dong et al. 2021). Additional research has shown that the body size of macrobenthos individuals within a species increases as they approach a pollution source (Ryu et al. 2011). Nevertheless, few studies have indicated that individual size is inversely correlated with the intensity of pollution; i.e., the higher the level of contamination, the smaller the individuals (Piló et al. 2016; Edegbene et al. 2020).

Feeding features convey information about an ecosystem’s ecological status and structural complexity (Tselepides et al. 2000; Bremner et al. 2003; Törnroos and Bonsdorff 2012). In the present study, detritus feeder was the most prevalent feature at highly contaminated sites (El-Mahmoudia Canal). While at less-contaminated sites (El-Nubaria Canal), scrapers were the most pervasive feature. In contrast, the scavenger carnivores and predators were the predominant feeding modes in non-contaminated locations; this could result from higher sediment quality, which allows organisms with diverse feeding habits to increase (Rosenberg 2001; Gusmao et al. 2016). Gaston et al. (1998) and Rakocinski et al. (2000) observed that subsurface deposit feeders predominated in areas with high organic and metal concentrations. In contrast, the number of members of other trophic groups, including carnivores, was low. According to Rawer-Jost et al. (2000) and Erasmus et al. (2021), anthropogenic influences cause a decrease in the number of scrapers.

Mobility of taxa and environmental disruptions were strongly associated (Nasi et al. 2018). In our study, mobility feature altered from burrowers at highly contaminated sites to climber and swimmers at less-contaminated sites and non-contaminated sites, respectively; this is favorable for the macrobenthos because it broadens their range of activity, increases the availability of food, and helps them quickly avoid polluted sediments (Dong et al. 2021).

This gradient and variation in feeding and mobility features in different sectors of the study area demonstrated that the trait-based method could evaluate the variation in the ecological status of the river ecosystem.

### Environmental driving factors analysis

The environmental variables influencing macrobenthic organisms are very complicated, and the associations between environmental factors and community structure differ between locations (Zhang et al. 2021). The richness of highly tolerant taxa increased from Sites N1 and B2 towards Sites B6 and B9 with cumulative nutrient (NH_4_, NO_2_, PO_4_) and metal (Na, Ca, Cd, and Pb) concentrations, signifying that each nutrient and metal affected the community structure.

In this study, RDA revealed that Na, DO, SiO_4_, and pH were the most influential environmental parameters in the macrobenthic community. Clements and Kotalik (2016) reported that anthropogenic disturbances, including industrial and agricultural discharges, are the sources of high concentrations of Na and Ca in the river ecosystems. Lethal effects of significant ions on aquatic macrobenthic are highly varied but mostly connected with increased ionic pressure and the metabolic cost associated with osmoregulation (Williams et al. 1999; Lob and Silver 2012; Cormier et al. 2013). Dissolved oxygen is one of the most significant vital abiotic influences on the aquatic ecosystem. The solubility and availability of nutrients are affected by dissolved oxygen; releasing nutrients from sediments accelerated under hypoxia and hence influences the production of aquatic ecosystems (Abdelmongy and El-Moselhy 2015). Ekau et al. (2010) recorded that the variations in dissolved oxygen concentrations affected the persistence of certain macrobenthic species, consequently on the species composition in the ecosystem. According to the present results, the study area recorded a widespread oxygen variation representing high hypoxia at site B9 (1.8 mg/l) during the autumn as a result of low water levels, in addition to untreated sewage, agricultural, and thermal discharge to high oxygen levels at site N8 (10.9 mg/l) in summer. This significant range in oxygen concentration in the water column has an immediate impact on the oxygenation procedure in the essential sediment deposit, resulting in macrobenthos devastations (Belal et al. 2016). In our study, oligochaetes were generally negatively correlated with DO concentrations. Lim et al. (2006) reported that oligochaetes were the most adaptable under anoxia. The positive correlation between *Micronecta* sp. and DO attention revealed the sensitivity of *Micronecta* sp. to pollution.

Because *Micronecta* sp. breathe DO and do not come up for air, they cannot survive in oxygen-depleted waters and are severely impacted by pollution (Gogala 2009). de Santiago et al. (2020) reported that silicate is the most significant nutrient-influenced macrobenthic community composition. Silica is abundant and constantly prevalent in surface water as dissolved, suspended, and colloidal forms. Dissolved formulae primarily consist of silica acid (El Sayed et al. 2020). Recent studies recorded a linear statistical association between the density of macrobenthos and silicate (Karlson et al. 2007; Ekeroth et al. 2016). pH is the crucial factor for the water criteria and organisms in the aquatic ecosystem (Goher et al. 2014) as it regulates the metal solubility and influences the natural environment. pH range (6.0–9.0) is the potential value for most aquatic organisms, but pH value (7) is the optimum value of the organism’s activity (Chin 2000). Many studies confirmed the significance of pH on macrobenthic organisms’ distribution and composition (Corfield 2000; Tripole et al. 2006; Almagro-Pastor et al. 2015).

The RDA model demonstrated that the distribution of trait categories was significantly influenced by calcium and cadmium. Oug et al. (2012) said that increased cadmium levels are associated with ecological functioning changes. In our study at high cadmium concentrations, trait features such as burrow-dwelling, mud substratum affinity, burrower, detritus feeders, large body size, and very tolerant pollution were predominant. In contrast, at low levels, free living, bentonic larval development, climber mobility, and small body size were characteristic. Leung and Tam (2013) stated that high Cd concentration is negatively associated with the macrobenthos community. Cadmium is one of the maximum significant heavy metals that may cause alterations in the functional traits of macrobenthos communities (Hu et al. 2019). In general, taxonomic and functional trait–based techniques explained that the significant factors responsible for the fluctuations in the macroinvertebrate community structure were the water chemistry variables, especially the metal concentrations. The taxonomic-based method demonstrated an apparent variance between diverse stressors (sewage, agriculture, and thermal effluents). The functional-based process discriminated a clear difference in the ecological status of contaminated sectors. Therefore, the integration between taxonomic and functional diversity can be a technique for assessing the environmental threat in a holistic sight (Ferreira et al. 2012).

## Conclusion

The current study on macrobenthic communities in El-Behery and El-Nassery rayahs recorded that the BTA approach perceived specific functional traits associated with pollution gradients and environmental variables. In the northern portion of El-Rayah El-Behery (El-Mahmoudia Canal), the distribution and trait patterns of the macrobenthic fauna indicated a significant level of eutrophication, where the primary functional features of the macrobenthic fauna shifted from small body size, carnivores, crawlers, swimmers, and pollution-intolerant species to large body size, detritus feeders, burrowers, and pollution-tolerant species. The traits “body size,” “feeding mode,” “mobility,” “habitat,” and “pollution tolerance” were identified as potential markers of the response of the macrobenthic fauna to the contaminations in the irrigation rayahs. The environmental parameters impacting macrobenthos’ structural and functional composition in irrigation canals were Na, DO, SiO_4_, pH, Ca, and cadmium. Human activities and uncontrolled sewage, industrial, and agricultural waste in the study area affected the taxonomic and trait compositions of the macrobenthos. Thus, enhancing the macrobenthos communities will require improved watershed ecology and pollution control.

## Supplementary Information

Below is the link to the electronic supplementary material.

Supplementary file1 (DOCX 96 KB)

## Data Availability

All data will be available from the corresponding author upon request.

## Code availability

Non-applicable.
