# Supplementary material for: Taxonomic and functional structure of macrobenthic invertebrate communities and their response to environmental variables along the subbranches of the Nile River (rayahs), Egypt
Source: Environ Sci Pollut Res Int. 2022 Nov 19;30(11):28803–17. doi: 10.1007/s11356-022-24140-z (PMC9995531; doi:10.1007/s11356-022-24140-z)
Supplement: Supplementary file 1 — Supplementary file1 (DOCX 96 KB) [file 11356_2022_24140_MOESM1_ESM.docx]

**Taxonomic and functional structure of macrobenthic invertebrate communities and their response to environmental variables along the subbranches of the Nile River (rayahs), Egypt**

Reda E. Bendary ^a^ *, Shaimaa M. Ibrahim ^a^, Mohamed E. Goher ^a^, Hosam E. Elsaied ^a^, Gamal M. El Shabrawy ^a^, Mohamed Abd El Mordy ^b^, Magdy T. Khalil ^b^

^a^ National Institute of Oceanography and Fisheries (NIOF), Cairo, Egypt.

^b^ Ain Shams University, Department of Zoology, Faculty of Science, Cairo, Egypt

*Corresponding author. E-mail address: [**rh.bendary@niof.sci.eg**](mailto:rh.bendary@niof.sci.eg)

R. E. Bendary Hydrobiology Lab., Freshwater & Lakes Division, National Institute of Oceanography and Fisheries (NIOF), Cairo, Egypt [rh.bendary@niof.sci.eg](mailto:rh.bendary@niof.sci.eg). ORCID ID **0000-0002-9161-2162; S**. M. Ibrahim Hydrobiology Lab., Freshwater & Lakes Division, National Institute of Oceanography and Fisheries (NIOF), Cairo, Egypt [Sm.ibrahem@niof.sci.eg](mailto:Sm.ibrahem@niof.sci.eg) ORCID ID 0000-0001-8688-3745; M. E. Goher Chemistry Lab., Freshwater & Lakes Division, National Institute of Oceanography and Fisheries (NIOF), Cairo, Egypt [smgoher@yahoo.com](mailto:smgoher@yahoo.com), ORCID ID [0000-0001-9377-2429](https://orcid.org/0000-0001-9377-2429); H. E. Elsaied Genetics Lab., Aquaculture Division, National Institute of Oceanography and Fisheries (NIOF), Cairo, Egypt [hosameasa@yahoo.com](mailto:hosameasa@yahoo.com) ORCID ID 0000-0002-6290-4852; G. M. El Shabrawy Hydrobiology Lab., Freshwater & Lakes Division, National Institute of Oceanography and Fisheries (NIOF), Cairo, Egypt [elshabrawy_gamal@yahoo.com](mailto:elshabrawy_gamal@yahoo.com). ORCID ID 0000-0002-7018-594X; M. Abd El Mordy, Department of Zoology, Faculty of Science, Ain Shams University, Cairo, Egypt mohamedmordy51@gmail. Com; M. T. Khalil, Department of Zoology, Faculty of Science, Ain Shams University, Cairo, Egypt [mtkhalil52@hotmail.com](mailto:mtkhalil52@hotmail.com) ORCID ID 0000-0002-5445-9437

**ST1 water Analysis**

The water samples were kept in polyethylene bottles in an ice box and analyzed in a laboratory following standard methods of the American Public Health Association **(APHA, 2005).** Total dissolved solids (TDS) were determined by evaporating a known volume of the filtrated sample at 180 °C. Dissolved oxygen (DO) was measured using the modified Winkler method. The COD was carried out using the potassium permanganate method... Concentrations of NO_2_-N, NH_4_-N, PO_4_-P, and SiO_4_ were determined using the colourimetric techniques with the formation of a reddish purple azo-dye, phenate, ascorbic acid molybdate, and molybdosilicate methods, respectively. Chloride (Cl) and sulphate (SO_4)_ were quantified using Mohr's and turbidimetric methods, respectively. Calcium (Ca) was determined via direct titration using an EDTA solution. Sodium (Na) and potassium (K) were determined using the flame photometer Model "Jenway PFP, United Kingdom". Heavy metals (Cd and Pb) were measured using an atomic absorption reader (SavantAA-AAS with GF 5000 Graphite Furnace) according to **APHA (2005)** after HNO_3_ digestion.

**References**

**APHA-American Public Health Association (2005)** Standard methods for the examination of water and wastewater. 21^th^ ed., 1015 pp, AWWA, WCPF, Washington DC

**Table S1** Environmental variables (mean ± SD) for the spring season at the sampling locations.

|  | **Temp**  **^o^C** | **TDS**  **mg/l** | **pH** | **DO**  **mg/l** | **COD**  **mg/l** | **NH_4_**  **µg/l** | **NO_2_**  **µg/l** | **PO_4_**  **µg/l** | **SiO_4_**  **mg/l** | **Cl**  **mg/l** | **Ca**  **mg/l** | **SO_4_**  **mg/l** | **Na**  **mg/l** | **K**  **mg/l** | **Pb**  **µg/l** | **Cd**  **µg/l** |
| --- | --- | --- | --- | --- | --- | --- | --- | --- | --- | --- | --- | --- | --- | --- | --- | --- |
| **B1** | **27.60** | **231.47** | **8.36** | **7.42** | **5.88** | **67.65** | **0.00** | **26.92** | **1.64** | **21.68** | **17.36** | **11.72** | **20.76** | **9.24** | **4.4** | **0.64** |
| **B2** | **26.50** | **234.51** | **8.32** | **7.97** | **5.12** | **327.64** | **5.01** | **16.83** | **3.40** | **22.78** | **17.48** | **11.78** | **22.19** | **9.9** | **12.8** | **0.41** |
| **B3** | **26.80** | **240.76** | **8.29** | **8.40** | **6.98** | **315.89** | **4.93** | **9.92** | **3.50** | **22.62** | **18.10** | **13.55** | **23.65** | **9.68** | **24** | **0.89** |
| **B4** | **26.60** | **241.12** | **8.46** | **7.87** | **5.76** | **302.44** | **3.89** | **22.32** | **3.45** | **22.36** | **18.40** | **15.16** | **24.57** | **9.02** | **20.6** | **0.8** |
| **B5** | **28.30** | **241.86** | **8.15** | **7.62** | **6.12** | **243.70** | **10.26** | **15.94** | **2.97** | **23.32** | **18.20** | **15.33** | **24.83** | **9.02** | **15.6** | **0.76** |
| **B6** | **27.40** | **251.11** | **8.27** | **7.96** | **5.44** | **389.61** | **9.94** | **13.11** | **2.84** | **23.38** | **18.94** | **16.04** | **25.11** | **9.24** | **11.2** | **1.12** |
| **B7** | **27.20** | **301.92** | **8.29** | **7.88** | **5.26** | **433.01** | **15.01** | **13.28** | **3.13** | **36.92** | **21.16** | **21.79** | **30.48** | **11** | **11.6** | **0.8** |
| **B8** | **25.80** | **334.16** | **8.30** | **8.34** | **14.22** | **331.81** | **18.14** | **11.51** | **2.83** | **38.54** | **22.62** | **22.42** | **34.37** | **11.56** | **21.4** | **1.4** |
| **B9** | **NC** | **NC** | **NC** | **NC** | **NC** | **NC** | **NC** | **NC** | **NC** | **NC** | **NC** | **NC** | **NC** | **NC** | **NC** | **NC** |
| **N1** | **27.60** | **231.47** | **8.36** | **7.42** | **5.88** | **67.65** | **0.00** | **26.92** | **1.64** | **21.68** | **17.36** | **11.72** | **20.76** | **9.24** | **13.4** | **0.45** |
| **N2** | **26.50** | **234.51** | **8.32** | **7.97** | **5.12** | **327.64** | **5.01** | **16.83** | **3.40** | **22.78** | **17.48** | **11.78** | **22.19** | **9.90** | **18.8** | **0.76** |
| **N3** | **26.80** | **240.76** | **8.29** | **8.40** | **6.98** | **315.89** | **4.93** | **9.92** | **3.50** | **22.62** | **18.10** | **13.55** | **23.65** | **9.68** | **7.4** | **0.79** |
| **N4** | **26.60** | **241.12** | **8.46** | **7.87** | **5.76** | **302.44** | **3.89** | **22.32** | **3.45** | **22.36** | **18.40** | **15.16** | **24.57** | **9.02** | **27.2** | **1.042** |
| **N5** | **28.30** | **241.86** | **8.15** | **7.62** | **6.12** | **243.70** | **10.26** | **15.94** | **2.97** | **23.32** | **18.20** | **15.33** | **24.83** | **9.02** | **19** | **0.87** |
| **N6** | **27.40** | **251.11** | **8.27** | **7.96** | **5.44** | **389.61** | **9.94** | **13.11** | **2.84** | **23.38** | **18.94** | **16.04** | **25.11** | **9.24** | **24.4** | **1.14** |
| **N7** | **27.20** | **301.92** | **8.29** | **7.88** | **5.26** | **433.01** | **15.01** | **13.28** | **3.13** | **36.92** | **21.16** | **21.79** | **30.48** | **11.00** | **24.8** | **1.32** |
| **N8** | **25.80** | **334.16** | **8.30** | **8.34** | **14.22** | **331.81** | **18.14** | **11.51** | **2.83** | **38.54** | **22.62** | **22.42** | **34.37** | **11.56** | **24.4** | **1.65** |
| **Minimum** | **25.8** | **231.47** | **8.15** | **7.42** | **5.12** | **67.65** | **0** | **9.92** | **1.64** | **21.68** | **17.36** | **11.72** | **20.76** | **9.02** | **4.4** | **0.41** |
| **Maximum** | **28.3** | **334.16** | **8.46** | **8.4** | **14.22** | **433.01** | **18.14** | **26.92** | **3.5** | **38.54** | **22.62** | **22.42** | **34.37** | **11.56** | **27.2** | **1.65** |
| **Mean** | **27.0** | **266.0** | **8.3** | **8.0** | **7.5** | **297.7** | **9.0** | **16.2** | **2.9** | **27.4** | **19.3** | **16.4** | **26.5** | **10.0** | **17.6** | **1.0** |
| **Std** | **0.8** | **39.5** | **0.1** | **0.3** | **3.4** | **103.3** | **6.2** | **5.5** | **0.6** | **7.1** | **1.9** | **4.1** | **4.8** | **1.0** | **6.7** | **0.4** |

**NC: Not collected**

**Table S2** Environmental variables (mean ± SD) for the summer season at the sampling locations.

|  | **Temp**  **^o^C** | **TDS**  **mg/l** | **pH** | **DO**  **mg/l** | **COD**  **mg/l** | **NH_4_**  **µg/l** | **NO_2_**  **µg/l** | **PO_4_**  **µg/l** | **SiO_4_**  **mg/l** | **Cl**  **mg/l** | **Ca**  **mg/l** | **SO_4_**  **mg/l** | **Na**  **mg/l** | **K**  **mg/l** | **Pb**  **µg/l** | **Cd**  **µg/l** |
| --- | --- | --- | --- | --- | --- | --- | --- | --- | --- | --- | --- | --- | --- | --- | --- | --- |
| **B1** | **29.6** | **243.2** | **8.6** | **6.8** | **9.3** | **82.8** | **2.0** | **15.5** | **2.8** | **20.2** | **21.6** | **12.7** | **22.6** | **8.3** | **20.2** | **0.6** |
| **B2** | **29.6** | **243.2** | **8.5** | **7.5** | **9.8** | **121.5** | **2.0** | **87.9** | **5.4** | **22.4** | **21.6** | **12.2** | **22.7** | **8.4** | **15.6** | **0.4** |
| **B3** | **30.2** | **243.9** | **8.5** | **8.0** | **7.6** | **82.2** | **2.0** | **78.9** | **2.9** | **23.1** | **22.3** | **12.3** | **22.7** | **8.7** | **24.0** | **1.0** |
| **B4** | **30.5** | **242.5** | **8.6** | **7.6** | **13.8** | **108.2** | **1.9** | **62.6** | **4.0** | **24.1** | **22.4** | **14.0** | **22.6** | **9.2** | **25.2** | **1.1** |
| **B5** | **31.5** | **243.9** | **8.6** | **6.9** | **16.3** | **281.2** | **8.5** | **79.0** | **1.4** | **25.5** | **23.1** | **13.0** | **24.1** | **8.4** | **19.6** | **1.1** |
| **B6** | **31.6** | **330.7** | **8.6** | **6.6** | **29.1** | **1070.5** | **142.7** | **78.3** | **4.1** | **34.4** | **30.1** | **17.2** | **34.9** | **10.3** | **29.2** | **1.4** |
| **B7** | **33.2** | **330.0** | **8.6** | **4.4** | **36.9** | **1178.9** | **117.7** | **87.1** | **3.4** | **35.3** | **31.1** | **18.4** | **34.6** | **10.3** | **30.1** | **1.0** |
| **B8** | **32.5** | **330.0** | **8.6** | **3.6** | **26.0** | **892.9** | **110.2** | **76.1** | **1.9** | **35.3** | **31.1** | **17.2** | **34.6** | **10.4** | **22.8** | **1.2** |
| **B9** | **29.8** | **331.4** | **8.7** | **2.9** | **26.0** | **534.6** | **65.5** | **119.7** | **2.9** | **36.4** | **32.7** | **16.9** | **33.5** | **10.5** | **27.2** | **1.2** |
| **N1** | **29.1** | **244.6** | **8.6** | **6.6** | **19.5** | **171.5** | **2.5** | **14.9** | **3.6** | **21.5** | **20.2** | **10.9** | **23.6** | **7.6** | **26.8** | **0.5** |
| **N2** | **30.0** | **244.6** | **8.5** | **6.8** | **7.6** | **152.0** | **2.8** | **79.7** | **5.0** | **22.3** | **21.1** | **10.4** | **23.7** | **7.8** | **13.8** | **1.0** |
| **N3** | **30.4** | **244.6** | **8.5** | **6.9** | **10.8** | **171.0** | **0.6** | **26.6** | **4.7** | **21.6** | **20.3** | **10.7** | **24.5** | **9.2** | **11.8** | **1.3** |
| **N4** | **30.9** | **243.9** | **8.5** | **6.9** | **11.9** | **140.6** | **0.3** | **19.5** | **5.4** | **21.3** | **22.3** | **10.5** | **23.4** | **8.6** | **14.4** | **0.9** |
| **N5** | **33.0** | **244.6** | **8.5** | **6.4** | **9.8** | **79.8** | **0.6** | **47.8** | **5.1** | **22.2** | **22.1** | **12.3** | **24.3** | **8.6** | **11.2** | **0.3** |
| **N6** | **32.5** | **246.0** | **8.5** | **6.5** | **15.2** | **119.7** | **0.0** | **19.5** | **3.9** | **21.5** | **21.6** | **11.9** | **23.1** | **8.5** | **16.4** | **0.9** |
| **N7** | **31.9** | **249.5** | **8.5** | **7.8** | **13.0** | **115.9** | **0.0** | **30.1** | **4.9** | **23.3** | **22.0** | **12.0** | **24.0** | **8.5** | **13.6** | **1.4** |
| **N8** | **30.4** | **249.5** | **8.4** | **10.9** | **12.4** | **102.6** | **5.7** | **31.9** | **3.7** | **24.1** | **21.0** | **12.4** | **25.6** | **8.4** | **14.8** | **1.5** |
| **Minimum** | **29.1** | **242.5** | **8.4** | **2.9** | **7.6** | **79.8** | **0.0** | **14.9** | **1.4** | **20.2** | **20.2** | **10.4** | **22.6** | **7.6** | **11.2** | **0.3** |
| **Maximum** | **33.2** | **331.4** | **8.7** | **10.9** | **36.9** | **1178.9** | **142.7** | **119.7** | **5.4** | **36.4** | **32.7** | **18.4** | **34.9** | **10.5** | **30.1** | **1.5** |
| **Mean** | **31.0** | **267.4** | **8.6** | **6.7** | **16.8** | **350.8** | **32.0** | **57.3** | **3.8** | **25.9** | **24.2** | **13.4** | **26.4** | **8.9** | **19.9** | **1.0** |
| **Std** | **1.3** | **37.5** | **0.1** | **1.8** | **8.5** | **367.9** | **48.8** | **32.4** | **1.2** | **5.7** | **4.3** | **2.6** | **4.8** | **0.9** | **6.4** | **0.4** |

**Table S3** Environmental variables (mean ± SD) for the autumn season at the sampling locations.

|  | **Temp**  **^o^C** | **TDS**  **mg/l** | **pH** | **DO**  **mg/l** | **COD**  **mg/l** | **NH_4_**  **µg/l** | **NO_2_**  **µg/l** | **PO_4_**  **µg/l** | **SiO_4_**  **mg/l** | **Cl**  **mg/l** | **SO_4_**  **mg/l** | **Ca**  **mg/l** | **Na**  **mg/l** | **K**  **mg/l** | **Pb**  **µg/l** | **Cd**  **µg/l** |
| --- | --- | --- | --- | --- | --- | --- | --- | --- | --- | --- | --- | --- | --- | --- | --- | --- |
| **B1** | **22.70** | **284.2** | **8.48** | **7.78** | **6.85** | **42.83** | **5.38** | **23.20** | **1.3** | **26.3** | **11.3** | **25.3** | **25.18** | **8.23** | **34.4** | **0.67** |
| **B2** | **23.00** | **284.9** | **8.35** | **6.67** | **7.89** | **44.91** | **5.07** | **14.17** | **1.1** | **26.3** | **10.7** | **25.3** | **25.75** | **8.34** | **21.4** | **0.58** |
| **B3** | **22.90** | **280.8** | **8.23** | **6.68** | **8.28** | **31.27** | **10.45** | **19.66** | **1.2** | **26.6** | **14.1** | **25.7** | **26.12** | **8.45** | **29.8** | **0** |
| **B4** | **22.70** | **287.6** | **8.72** | **7.31** | **7.78** | **154.82** | **22.00** | **16.47** | **2.4** | **28.5** | **11.9** | **26.6** | **26.75** | **8.53** | **24.2** | **0.67** |
| **B5** | **22.60** | **286.9** | **8.42** | **6.09** | **7.57** | **309.26** | **18.43** | **31.17** | **1.1** | **30.0** | **15.2** | **27.1** | **28.29** | **8.52** | **36.4** | **0.86** |
| **B6** | **22.30** | **445.5** | **8.24** | **5.04** | **16.97** | **3115.17** | **447.81** | **129.30** | **3.6** | **46.3** | **23.2** | **40.6** | **47** | **10.83** | **31.8** | **1.47** |
| **B7** | **24.00** | **418.7** | **7.92** | **3.60** | **15.89** | **3045.82** | **407.01** | **166.15** | **1.7** | **44.8** | **23.3** | **39.5** | **43.95** | **10.05** | **28.8** | **1.18** |
| **B8** | **23.80** | **422.1** | **7.85** | **3.36** | **15.01** | **2356.04** | **380.10** | **113.89** | **1.4** | **45.1** | **21.9** | **39.8** | **44.31** | **10.29** | **59.6** | **1.12** |
| **B9** | **22.20** | **413.2** | **7.64** | **1.83** | **19.66** | **1913.18** | **268.47** | **95.30** | **4.5** | **45.4** | **21.1** | **40.8** | **41.75** | **10.03** | **52** | **1.31** |
| **N1** | **22.30** | **284.2** | **8.11** | **8.12** | **7.72** | **38.09** | **18.05** | **26.21** | **3.8** | **25.0** | **12.7** | **23.5** | **27.42** | **8.86** | **35.6** | **0.67** |
| **N2** | **22.40** | **280.1** | **8.26** | **7.69** | **9.69** | **36.00** | **8.86** | **18.42** | **2.2** | **25.6** | **11.9** | **24.1** | **27.16** | **8.82** | **35.4** | **0.72** |
| **N3** | **23.00** | **279.5** | **8.48** | **5.80** | **8.46** | **56.66** | **16.25** | **16.83** | **2.4** | **24.7** | **12.2** | **23.1** | **27.99** | **8.56** | **43.86** | **0.67** |
| **N4** | **22.50** | **285.6** | **8.48** | **5.69** | **7.39** | **44.20** | **6.32** | **34.10** | **3.9** | **24.9** | **12.3** | **26.1** | **27.35** | **8.04** | **46** | **0.46** |
| **N5** | **25.10** | **292.4** | **8.18** | **7.10** | **8.64** | **66.30** | **3.16** | **11.00** | **3.3** | **26.5** | **14.8** | **26.4** | **29.06** | **8.25** | **33.2** | **0.82** |
| **N6** | **24.10** | **285.0** | **8.39** | **6.58** | **8.07** | **100.30** | **3.48** | **12.10** | **3.5** | **24.9** | **13.7** | **25.0** | **26.78** | **8.86** | **33.8** | **1.28** |
| **N7** | **23.30** | **299.4** | **8.37** | **6.76** | **8.27** | **57.80** | **8.22** | **30.80** | **4.1** | **28.0** | **14.4** | **21.6** | **28.81** | **9.25** | **36.6** | **1.06** |
| **N8** | **22.90** | **411.5** | **8.81** | **8.10** | **7.58** | **144.50** | **6.64** | **27.50** | **3.3** | **39.7** | **20.5** | **29.7** | **42.27** | **10.53** | **29.6** | **2.07** |
| **Minimum** | **22.2** | **279.5** | **7.6** | **1.8** | **6.9** | **31.3** | **3.2** | **11.0** | **1.1** | **24.7** | **10.7** | **21.6** | **25.2** | **8.0** | **21.4** | **0.0** |
| **Maximum** | **25.1** | **445.5** | **8.8** | **8.1** | **19.7** | **3115.2** | **447.8** | **166.2** | **4.5** | **46.3** | **23.3** | **40.8** | **47.0** | **10.8** | **59.6** | **2.1** |
| **Mean** | **23.1** | **329.8** | **8.3** | **6.0** | **10.4** | **773.9** | **109.8** | **50.7** | **2.7** | **32.1** | **29.1** | **15.8** | **32.5** | **9.1** | **36.5** | **0.9** |
| **Std.** | **0.8** | **64.6** | **0.3** | **1.8** | **4.0** | **1132.1** | **163.4** | **48.0** | **1.2** | **8.6** | **6.7** | **4.5** | **7.9** | **0.9** | **9.7** | **0.5** |

**Table S4** Environmental variables (mean ± SD) for the winter season at the sampling locations

|  | **Temp**  **^o^C** | **TDS**  **mg/l** | **pH** | **DO**  **mg/l** | **COD**  **mg/l** | **NH_4_**  **µg/l** | **NO_2_**  **µg/l** | **PO_4_**  **µg/l** | **SiO_4_**  **mg/l** | **Cl**  **mg/l** | **SO_4_**  **mg/l** | **Ca**  **mg/l** | **Na**  **mg/l** | **K**  **mg/l** | **Pb**  **µg/l** | **Cd**  **µg/l** |
| --- | --- | --- | --- | --- | --- | --- | --- | --- | --- | --- | --- | --- | --- | --- | --- | --- |
| **B1** | **19.50** | **273.94** | **8.10** | **8.58** | **6.96** | **200.68** | **22.16** | **23.20** | **1.40** | **25.37** | **12.87** | **22.38** | **24.28** | **7.93** | **34.6** | **0.53** |
| **B2** | **19.60** | **276.67** | **8.05** | **8.15** | **7.76** | **169.79** | **12.54** | **14.17** | **1.56** | **25.52** | **13.43** | **22.61** | **25.01** | **8.1** | **40.2** | **0.62** |
| **B3** | **19.90** | **278.74** | **8.11** | **6.40** | **8.53** | **261.70** | **10.92** | **19.66** | **1.43** | **26.42** | **13.04** | **23.53** | **25.93** | **7.89** | **20.6** | **0.16** |
| **B4** | **15.10** | **302.61** | **8.13** | **9.59** | **8.28** | **144.21** | **7.85** | **16.47** | **1.37** | **26.02** | **13.48** | **25.95** | **25.15** | **8.29** | **26.4** | **0.83** |
| **B5** | **15.90** | **306.75** | **7.92** | **8.71** | **1.86** | **208.45** | **8.29** | **31.17** | **1.71** | **26.06** | **13.30** | **26.99** | **26.24** | **8.45** | **12** | **0.96** |
| **B6** | **16.00** | **637.34** | **7.91** | **5.25** | **18.42** | **6733.48** | **60.06** | **129.30** | **1.58** | **62.29** | **31.19** | **40.51** | **59.23** | **10.42** | **47.6** | **1.35** |
| **B7** | **18.60** | **529.38** | **8.12** | **5.76** | **16.68** | **6438.98** | **95.32** | **166.15** | **1.43** | **50.67** | **29.46** | **40.94** | **49.57** | **9.88** | **42.8** | **1.12** |
| **B8** | **18.10** | **554.13** | **7.72** | **5.12** | **14.20** | **6675.67** | **155.38** | **113.89** | **1.91** | **53.21** | **29.81** | **40.27** | **48.17** | **9.97** | **37.2** | **1.04** |
| **B9** | **19.50** | **605.05** | **7.41** | **4.80** | **19.48** | **6255.36** | **231.62** | **95.30** | **1.27** | **58.49** | **30.92** | **47.77** | **57.14** | **10.08** | **42.2** | **1.22** |
| **N1** | **18.50** | **280.13** | **8.11** | **8.32** | **8.04** | **202.20** | **11.68** | **4.96** | **1.72** | **24.66** | **12.53** | **19.16** | **27.03** | **7.73** | **37.4** | **0.84** |
| **N2** | **19.80** | **286.28** | **8.21** | **7.34** | **9.52** | **248.05** | **12.19** | **6.91** | **1.54** | **26.14** | **12.19** | **22.65** | **27.76** | **7.01** | **27.6** | **0.72** |
| **N3** | **18.60** | **287.64** | **8.26** | **6.63** | **10.76** | **280.46** | **11.27** | **7.62** | **1.70** | **26.54** | **12.44** | **22.43** | **28.18** | **7.19** | **35.2** | **0.59** |
| **N4** | **16.10** | **299.24** | **8.17** | **7.96** | **8.84** | **47.60** | **15.20** | **2.23** | **1.36** | **26.08** | **12.89** | **22.36** | **28.66** | **7.43** | **31** | **1.07** |
| **N5** | **17.80** | **300.63** | **8.02** | **9.12** | **9.08** | **66.30** | **14.80** | **3.35** | **1.80** | **27.25** | **15.17** | **23.15** | **29.88** | **7.48** | **41** | **1.15** |
| **N6** | **18.60** | **310.92** | **8.18** | **9.08** | **8.88** | **141.10** | **16.00** | **4.19** | **1.48** | **27.21** | **14.99** | **23.28** | **29.23** | **7.66** | **26.2** | **0.75** |
| **N7** | **17.70** | **326.74** | **8.13** | **8.67** | **8.96** | **79.90** | **28.80** | **4.63** | **1.64** | **30.57** | **15.77** | **25.55** | **31.44** | **8.10** | **15** | **1.28** |
| **N8** | **15.00** | **505.84** | **8.14** | **8.90** | **8.64** | **297.50** | **34.80** | **6.70** | **1.76** | **48.79** | **25.20** | **34.54** | **51.97** | **10.94** | **22.4** | **1.38** |
| **Minimum** | **15.0** | **273.9** | **7.4** | **4.8** | **1.9** | **47.6** | **7.9** | **2.2** | **1.3** | **24.7** | **12.2** | **19.2** | **24.3** | **7.0** | **12.0** | **0.2** |
| **Maximum** | **19.9** | **637.3** | **8.3** | **9.6** | **19.5** | **6733.5** | **231.6** | **166.2** | **1.9** | **62.3** | **31.2** | **47.8** | **59.2** | **10.9** | **47.6** | **1.4** |
| **Mean** | **16.7** | **386.6** | **7.6** | **7.2** | **10.4** | **2179.5** | **58.7** | **47.6** | **1.5** | **36.1** | **28.9** | **18.8** | **36.2** | **8.2** | **30.3** | **0.9** |
| **Std.** | **1.7** | **140.1** | **0.3** | **1.7** | **5.3** | **2973.5** | **75.8** | **60.9** | **0.2** | **14.7** | **9.8** | **8.0** | **13.2** | **1.4** | **11.5** | **0.3** |

**Table S5** Total mean abundance (ind./m^2^) of taxonomic groups of macrobenthic communities in the study area.

| taxa | B1 | B2 | B3 | B4 | B5 | B6 | B7 | B8 | B9 | N1 | N2 | N3 | N4 | N5 | N6 | N7 | N8 |
| --- | --- | --- | --- | --- | --- | --- | --- | --- | --- | --- | --- | --- | --- | --- | --- | --- | --- |
| Insecta | 731 | 144 | 131 | 231 | 419 | 2744 | 163 | 406 | 181 | 213 | 869 | 694 | 138 | 181 | 438 | 231 | 681 |
| Crustacea | 0 | 0 | 0 | 0 | 6 | 0 | 0 | 6 | 0 | 0 | 31 | 19 | 0 | 0 | 0 | 0 | 44 |
| Gastropoda | 13 | 0 | 6 | 44 | 19 | 19 | 50 | 244 | 2106 | 25 | 350 | 75 | 100 | 88 | 100 | 88 | 100 |
| Bivalvia | 6 | 0 | 88 | 0 | 13 | 88 | 163 | 106 | 394 | 31 | 31 | 50 | 26 | 75 | 13 | 38 | 13 |
| Oligochaeta | 1157 | 331 | 1119 | 1569 | 4500 | 2413 | 1056 | 1213 | 2600 | 700 | 444 | 2400 | 1500 | 3563 | 1131 | 2800 | 1638 |
| Individuals | 1907 | 476 | 1344 | 1844 | 4956 | 5263 | 1431 | 1975 | 5281 | 969 | 1725 | 3238 | 1763 | 3906 | 1681 | 3156 | 2475 |
| species | 9 | 6 | 10 | 7 | 9 | 12 | 9 | 17 | 14 | 10 | 15 | 15 | 10 | 12 | 12 | 13 | 18 |

**Table S6** Average abundance (ind./m^2^) of the macrobenthic species during different seasons in the study area.

| **Taxa** | **spring** | **summer** | **autumn** | **winter** |
| --- | --- | --- | --- | --- |
| **Insecta** |  |  |  |  |
| **Chironomidae larva** | 619.1 | 111.8 | 270.6 | 488.2 |
| **Chironomidae pupa** | 17.6 | 13.2 | 8.8 | 11.8 |
| ***Micronecta* sp.** | 8.8 | 7.4 | 4.4 | 313.2 |
| ***Caenis* sp.** | 1.5 | 0.0 | 2.9 | 1.5 |
| ***Ischnura* sp.** | 2.9 | 0.0 | 0.0 | 8.8 |
| ***Coanagrion* sp.** | 0.0 | 0.0 | 1.5 | 0.0 |
| ***Cloenon* sp.** | 0.0 | 2.9 | 0.0 | 0.0 |
| ***Baetis* sp.** | 0.0 | 0.0 | 10.3 | 0.0 |
| ***Hydroptilidae larva*** | 0.0 | 52.9 | 38.2 | 5.9 |
| ***Orthetrum* sp*.*** | 0.0 | 0.0 | 0.0 | 10.3 |
| ***Philopotamus* sp.** | 0.0 | 0.0 | 0.0 | 1.5 |
| ***Hydropsychidae larva*** | 0.0 | 1.5 | 0.0 | 0.0 |
| ***Hyphoporus* sp.** | 0.0 | 0.0 | 0.0 | 1.5 |
| ***Lepidoptera larva*** | 0.0 | 0.0 | 0.0 | 1.5 |
| ***Tabanidae larva*** | 0.0 | 0.0 | 0.0 | 1.5 |
| ***Crustacea*** |  |  |  |  |
| ***Procambarus clarkia*** | 0.0 | 0.0 | 1.5 | 0.0 |
| ***Caridina nilotica*** | 1.5 | 1.5 | 1.5 | 17.6 |
| ***Isopoda* sp.** | 0.0 | 0.0 | 0.0 | 0.0 |
| ***Gastropoda*** |  |  |  |  |
| ***Theodoxus niloticus*** | 5.9 | 4.4 | 0.0 | 8.8 |
| ***Viviparus contectus*** | 1.5 | 19.1 | 5.9 | 5.9 |
| ***Cleopatra bulimoides*** | 8.8 | 20.6 | 5.9 | 30.9 |
| ***Melanoides tuberculata*** | 10.3 | 75.0 | 0.0 | 79.4 |
| ***Pila ovata*** | 1.5 | 0.0 | 0.0 | 0.0 |
| ***Gabbiella senaariensis*** | 11.8 | 473.5 | 2.9 | 16.2 |
| ***Bulinus truncates*** | 1.5 | 4.4 | 1.5 | 2.9 |
| ***Lanistes carinatus*** | 0.0 | 4.4 | 1.5 | 1.5 |
| ***Bivalvia*** |  |  |  |  |
| ***Mutela rostrata*** | 0.0 | 4.4 | 5.9 | 1.5 |
| ***Corbicula fluminalis*** | 8.8 | 101.5 | 11.8 | 70.6 |
| ***Coelatura aegyptiaca*** | 14.7 | 2.9 | 7.4 | 25.0 |
| ***Coelatura prasidens*** | 1.5 | 0.0 | 0.0 | 0.0 |
| ***Oligochaeta*** |  |  |  |  |
| ***Limnodrilus udekemianus*** | 1754.4 | 1533.8 | 1498.5 | 2027.9 |
| ***Branchiura sowerbyi*** | 0.0 | 60.3 | 16.2 | 44.1 |
| ***Allolobophora caliginosa*** | 0.0 | 2.9 | 1.5 | 1.5 |
| ***Helobdella conifer*** | 27.9 | 0.0 | 17.6 | 47.1 |
| ***Limnatis nilotica*** | 8.8 | 2.9 | 2.9 | 7.4 |
| ***Batracobdelloides* sp.** | 5.9 | 1.5 | 1.5 | 1.5 |
| ***Barbronia assiuti*** | 2.9 | 1.5 | 0.0 | 19.1 |

**Table S7** Fuzzy coded trait data of each taxon of benthic macroinvertebrates were recorded in El-Rayah El- Behery and El-Rayah El- Nassery.

| **Traits** | **Morphology** | | **Body size(mm)** | | | | **Longevity (year)** | | | | **Feeding mode** | | | | | | **Mobility** | | | | | |
| --- | --- | --- | --- | --- | --- | --- | --- | --- | --- | --- | --- | --- | --- | --- | --- | --- | --- | --- | --- | --- | --- | --- |
| **Species/code** | **M1** | **M2** | **S1** | **S2** | **S3** | **S4** | **L1** | **L2** | **L3** | **L4** | **F1** | **F2** | **F3** | **F4** | **F5** | **F6** | **mob1** | **mob2** | **mob3** | **mob4** | **mob5** | **mob6** |
| **Chironomidae larva** | **0** | **3** | **3** | **0** | **0** | **0** | **3** | **0** | **0** | **0** | **0** | **3** | **3** | **3** | **3** | **0** | **3** | **3** | **0** | **0** | **0** | **0** |
| **Chironomidae pupa** | **0** | **3** | **3** | **0** | **0** | **0** | **3** | **0** | **0** | **0** | **0** | **3** | **3** | **3** | **3** | **0** | **3** | **3** | **0** | **0** | **0** | **3** |
| ***Micronecta sp.*** | **0** | **3** | **3** | **0** | **0** | **0** | **3** | **0** | **0** | **0** | **0** | **0** | **0** | **0** | **0** | **3** | **0** | **0** | **0** | **0** | **0** | **3** |
| ***Caenis sp.*** | **0** | **3** | **3** | **3** | **0** | **0** | **3** | **0** | **0** | **0** | **0** | **0** | **3** | **3** | **0** | **0** | **0** | **0** | **3** | **0** | **3** | **0** |
| ***Ischnura sp.*** | **0** | **3** | **0** | **2** | **3** | **0** | **3** | **0** | **0** | **0** | **0** | **0** | **0** | **0** | **3** | **0** | **0** | **0** | **0** | **0** | **3** | **0** |
| ***Coanagrion sp.*** | **0** | **3** | **0** | **2** | **3** | **0** | **3** | **0** | **0** | **0** | **0** | **0** | **0** | **0** | **3** | **0** | **0** | **0** | **0** | **0** | **3** | **0** |
| ***Cloenon sp.*** | **0** | **3** | **3** | **0** | **0** | **0** | **3** | **0** | **0** | **0** | **0** | **0** | **0** | **3** | **0** | **0** | **0** | **3** | **0** | **0** | **0** | **3** |
| ***Baetis sp.*** | **0** | **3** | **2** | **3** | **0** | **0** | **3** | **0** | **0** | **0** | **0** | **0** | **0** | **3** | **0** | **0** | **0** | **3** | **0** | **0** | **0** | **3** |
| ***Hydroptilidae larva*** | **0** | **3** | **3** | **0** | **0** | **0** | **3** | **2** | **0** | **0** | **0** | **0** | **3** | **3** | **2** | **2** | **0** | **3** | **0** | **0** | **3** | **0** |
| ***Hyphoporus sp.*** | **0** | **3** | **0** | **0** | **3** | **0** | **3** | **0** | **0** | **0** | **0** | **0** | **0** | **0** | **3** | **0** | **0** | **3** | **0** | **0** | **0** | **0** |
| ***Orthetrum sp.*** | **0** | **3** | **0** | **3** | **0** | **0** | **3** | **0** | **0** | **0** | **0** | **0** | **0** | **0** | **3** | **0** | **3** | **0** | **0** | **0** | **0** | **0** |
| ***Philopotamus sp.*** | **0** | **3** | **3** | **0** | **0** | **0** | **3** | **0** | **0** | **0** | **0** | **0** | **3** | **0** | **3** | **0** | **0** | **0** | **0** | **0** | **0** | **0** |
| ***Lepidoptera larva*** | **0** | **3** | **3** | **0** | **0** | **0** | **3** | **0** | **0** | **0** | **0** | **3** | **2** | **2** | **2** | **2** | **0** | **0** | **0** | **3** | **0** | **0** |
| ***Tabanidae larva*** | **0** | **3** | **0** | **0** | **3** | **3** | **3** | **0** | **0** | **0** | **0** | **3** | **0** | **0** | **3** | **0** | **0** | **0** | **0** | **0** | **0** | **0** |
| ***Hydropsychidae larva*** | **0** | **3** | **0** | **3** | **0** | **0** | **3** | **0** | **0** | **0** | **0** | **0** | **0** | **0** | **3** | **0** | **0** | **3** | **0** | **0** | **0** | **0** |
| ***Caridina nilotica*** | **0** | **3** | **0** | **0** | **3** | **0** | **0** | **3** | **3** | **0** | **0** | **0** | **3** | **0** | **0** | **0** | **0** | **0** | **0** | **0** | **2** | **3** |
| ***Isopoda sp.*** | **0** | **3** | **3** | **0** | **0** | **0** | **3** | **3** | **0** | **0** | **0** | **3** | **3** | **3** | **3** | **3** | **0** | **0** | **3** | **3** | **2** | **3** |
| ***Procambarus clarkia*** | **0** | **3** | **0** | **0** | **0** | **3** | **0** | **3** | **3** | **0** | **0** | **3** | **3** | **0** | **3** | **3** | **3** | **0** | **0** | **0** | **0** | **3** |
| ***Theodoxus niloticus*** | **0** | **3** | **0** | **3** | **0** | **0** | **0** | **0** | **3** | **0** | **3** | **0** | **0** | **3** | **0** | **0** | **0** | **0** | **0** | **3** | **0** | **1** |
| ***Gabbiella senaariensis*** | **0** | **3** | **0** | **0** | **3** | **0** | **0** | **0** | **3** | **0** | **3** | **0** | **0** | **3** | **0** | **0** | **0** | **0** | **0** | **3** | **0** | **1** |
| ***Cleopatra bulimoides*** | **0** | **3** | **0** | **0** | **0** | **3** | **0** | **0** | **3** | **0** | **3** | **0** | **0** | **3** | **0** | **0** | **0** | **0** | **0** | **3** | **0** | **1** |
| ***Melanoides tuberculata*** | **0** | **3** | **0** | **0** | **0** | **3** | **0** | **0** | **3** | **0** | **3** | **0** | **0** | **3** | **0** | **0** | **0** | **0** | **0** | **3** | **0** | **1** |
| ***Viviparus contectus*** | **0** | **3** | **0** | **0** | **0** | **3** | **0** | **0** | **3** | **3** | **3** | **0** | **0** | **3** | **0** | **0** | **0** | **0** | **0** | **3** | **0** | **1** |
| ***Lanistes carinatus*** | **0** | **3** | **0** | **0** | **0** | **3** | **0** | **3** | **0** | **0** | **3** | **0** | **0** | **3** | **0** | **0** | **0** | **0** | **0** | **3** | **0** | **1** |
| ***Bulinus truncates*** | **0** | **3** | **0** | **0** | **3** | **0** | **0** | **0** | **3** | **0** | **3** | **0** | **0** | **0** | **0** | **0** | **0** | **0** | **0** | **3** | **0** | **1** |
| ***Corbicula fluminalis*** | **0** | **3** | **0** | **0** | **0** | **3** | **0** | **0** | **3** | **0** | **3** | **0** | **0** | **3** | **0** | **0** | **3** | **0** | **0** | **3** | **0** | **3** |
| ***Coelatura aegyptiaca*** | **0** | **3** | **0** | **0** | **0** | **3** | **0** | **0** | **0** | **3** | **3** | **0** | **0** | **0** | **0** | **0** | **3** | **0** | **0** | **0** | **0** | **3** |
| ***Coelatura prasidens*** | **0** | **3** | **0** | **0** | **0** | **3** | **0** | **0** | **0** | **3** | **3** | **0** | **0** | **0** | **0** | **0** | **3** | **0** | **0** | **0** | **0** | **3** |
| ***Mutela rostrata*** | **0** | **3** | **0** | **0** | **0** | **3** | **0** | **0** | **0** | **3** | **3** | **0** | **0** | **0** | **0** | **0** | **3** | **0** | **0** | **0** | **0** | **3** |
| ***pila ovata*** | **0** | **3** | **0** | **0** | **3** | **0** | **0** | **3** | **2** | **0** | **3** | **0** | **0** | **3** | **0** | **0** | **0** | **0** | **0** | **3** | **0** | **1** |
| ***Limnodrilus udekemianus*** | **3** | **0** | **0** | **0** | **0** | **3** | **0** | **0** | **3** | **0** | **0** | **3** | **3** | **0** | **0** | **0** | **3** | **0** | **0** | **0** | **0** | **0** |
| ***Branchiura sowerbyi*** | **3** | **0** | **0** | **0** | **3** | **3** | **0** | **2** | **3** | **0** | **0** | **3** | **3** | **0** | **0** | **0** | **3** | **0** | **0** | **0** | **0** | **0** |
| ***Helobdella conifer*** | **3** | **0** | **0** | **0** | **3** | **0** | **0** | **0** | **3** | **0** | **0** | **3** | **0** | **0** | **3** | **0** | **0** | **0** | **0** | **0** | **0** | **0** |
| ***Batracobdelloides sp.*** | **3** | **0** | **0** | **0** | **3** | **0** | **0** | **0** | **3** | **0** | **0** | **3** | **0** | **0** | **3** | **0** | **0** | **0** | **0** | **0** | **0** | **0** |
| ***Limnatis nilotica*** | **3** | **0** | **0** | **0** | **0** | **3** | **0** | **0** | **3** | **0** | **0** | **3** | **0** | **0** | **3** | **0** | **0** | **0** | **0** | **0** | **0** | **0** |
| ***Barbronia assiuti*** | **3** | **0** | **0** | **0** | **0** | **3** | **0** | **0** | **3** | **0** | **0** | **3** | **0** | **0** | **3** | **0** | **0** | **0** | **0** | **0** | **0** | **0** |
| ***Allolobophora caliginosa*** | **3** | **0** | **0** | **0** | **0** | **3** | **0** | **0** | **0** | **0** | **0** | **3** | **3** | **0** | **0** | **0** | **3** | **0** | **0** | **0** | **0** | **0** |

**Table S7** (continued) Fuzzy coded trait data of each taxon of benthic macroinvertebrates were recorded in El-Rayah El- Behery and El-Rayah El- Nassery.

***(NBPI) A Nile Biotic Pollution Index**

| **Traits** | **Habitat** | | | | **Substrate affinity** | | | | | **Reproductive technique** | | **Larval environmental development** | | | **Pollution tolerance (NBPI)** | | | |
| --- | --- | --- | --- | --- | --- | --- | --- | --- | --- | --- | --- | --- | --- | --- | --- | --- | --- | --- |
| **Species/code** | **H1** | **H2** | **H3** | **H4** | **SA1** | **SA2** | **SA3** | **SA4** | **SA5** | **R1** | **R2** | **LD1** | **LD2** | **LD3** | **P1** | **P2** | **P3** | **P4** |
| ***Chironomidae larva*** | **0** | **0** | **3** | **3** | **3** | **0** | **0** | **1** | **2** | **3** | **0** | **0** | **0** | **3** | **0** | **0** | **0** | **3** |
| ***Chironomidae pupa*** | **0** | **0** | **3** | **3** | **3** | **0** | **0** | **1** | **2** | **3** | **0** | **0** | **3** | **3** | **0** | **0** | **0** | **3** |
| ***Micronecta sp.*** | **0** | **3** | **3** | **0** | **0** | **0** | **0** | **0** | **3** | **3** | **0** | **0** | **0** | **0** | **3** | **0** | **0** | **0** |
| ***Caenis sp.*** | **0** | **0** | **3** | **3** | **3** | **0** | **0** | **1** | **2** | **3** | **0** | **0** | **3** | **2** | **0** | **0** | **3** | **0** |
| ***Ischnura sp.*** | **0** | **3** | **0** | **0** | **0** | **0** | **0** | **2** | **1** | **3** | **0** | **0** | **0** | **0** | **0** | **0** | **3** | **2** |
| ***Coanagrion sp.*** | **0** | **3** | **0** | **0** | **0** | **0** | **0** | **2** | **1** | **3** | **0** | **0** | **0** | **0** | **0** | **0** | **3** | **2** |
| ***Cloenon sp.*** | **0** | **0** | **3** | **2** | **2** | **0** | **0** | **0** | **2** | **3** | **0** | **0** | **0** | **0** | **3** | **0** | **0** | **0** |
| ***Baetis sp.*** | **0** | **0** | **3** | **2** | **2** | **0** | **0** | **0** | **2** | **3** | **0** | **0** | **0** | **0** | **0** | **3** | **2** | **0** |
| ***Hydroptilidae larva*** | **0** | **3** | **3** | **0** | **0** | **0** | **0** | **0** | **3** | **3** | **0** | **0** | **3** | **0** | **0** | **3** | **0** | **0** |
| ***Hyphoporus sp.*** | **0** | **0** | **2** | **3** | **3** | **0** | **0** | **0** | **2** | **3** | **0** | **0** | **2** | **3** | **0** | **3** | **0** | **0** |
| ***Orthetrum sp.*** | **0** | **3** | **0** | **0** | **0** | **0** | **0** | **0** | **2** | **3** | **0** | **0** | **3** | **2** | **0** | **0** | **3** | **3** |
| ***Philopotamus sp.*** | **0** | **0** | **3** | **3** | **3** | **0** | **3** | **3** | **0** | **3** | **0** | **0** | **3** | **2** | **0** | **3** | **0** | **0** |
| ***Lepidoptera larva*** | **0** | **0** | **0** | **3** | **3** | **0** | **0** | **0** | **2** | **3** | **0** | **0** | **3** | **1** | **0** | **3** | **0** | **0** |
| ***Tabanidae larva*** | **0** | **0** | **3** | **3** | **3** | **0** | **0** | **0** | **2** | **3** | **0** | **0** | **3** | **3** | **0** | **0** | **3** | **0** |
| ***Hydropsychidae larva*** | **0** | **0** | **0** | **3** | **3** | **3** | **0** | **0** | **0** | **3** | **0** | **0** | **3** | **0** | **0** | **3** | **0** | **0** |
| ***Caridina nilotica*** | **0** | **2** | **3** | **2** | **2** | **0** | **0** | **0** | **2** | **3** | **0** | **0** | **3** | **0** | **0** | **3** | **0** | **0** |
| ***Isopoda sp.*** | **0** | **0** | **0** | **3** | **3** | **3** | **2** | **0** | **0** | **3** | **1** | **3** | **0** | **0** | **0** | **0** | **3** | **0** |
| ***Procambarus clarkia*** | **0** | **3** | **0** | **0** | **0** | **2** | **2** | **3** | **3** | **3** | **0** | **3** | **0** | **0** | **0** | **0** | **3** | **0** |
| ***Theodoxus niloticus*** | **0** | **0** | **3** | **3** | **3** | **3** | **1** | **0** | **0** | **3** | **0** | **0** | **0** | **3** | **0** | **0** | **2** | **3** |
| ***Gabbiella senaariensis*** | **0** | **0** | **3** | **3** | **3** | **0** | **0** | **2** | **3** | **3** | **0** | **0** | **0** | **3** | **0** | **0** | **3** | **3** |
| ***Cleopatra bulimoides*** | **0** | **0** | **3** | **3** | **3** | **3** | **0** | **0** | **0** | **3** | **0** | **0** | **0** | **3** | **0** | **0** | **3** | **3** |
| ***Melanoides tuberculata*** | **0** | **0** | **3** | **3** | **3** | **3** | **0** | **0** | **0** | **3** | **0** | **0** | **0** | **3** | **0** | **0** | **3** | **3** |
| ***Viviparus contectus*** | **0** | **0** | **3** | **3** | **3** | **0** | **0** | **0** | **0** | **3** | **0** | **3** | **0** | **0** | **0** | **0** | **3** | **0** |
| ***Lanistes carinatus*** | **0** | **0** | **3** | **3** | **3** | **0** | **0** | **0** | **0** | **3** | **0** | **3** | **0** | **0** | **0** | **0** | **0** | **3** |
| ***Bulinus truncates*** | **0** | **0** | **3** | **3** | **3** | **0** | **0** | **0** | **0** | **3** | **0** | **3** | **0** | **0** | **0** | **0** | **3** | **0** |
| ***Corbicula fluminalis*** | **0** | **0** | **3** | **3** | **3** | **0** | **3** | **3** | **3** | **0** | **3** | **0** | **0** | **3** | **0** | **0** | **3** | **0** |
| ***Coelatura aegyptiaca*** | **3** | **0** | **0** | **0** | **0** | **3** | **3** | **3** | **0** | **3** | **0** | **0** | **0** | **3** | **0** | **3** | **0** | **0** |
| ***Coelatura prasidens*** | **3** | **0** | **0** | **0** | **0** | **3** | **3** | **3** | **0** | **3** | **0** | **0** | **0** | **3** | **0** | **3** | **0** | **0** |
| ***Mutela rostrata*** | **3** | **0** | **0** | **0** | **0** | **3** | **3** | **3** | **0** | **3** | **0** | **0** | **0** | **3** | **0** | **3** | **0** | **0** |
| ***pila ovata*** | **0** | **0** | **3** | **3** | **3** | **0** | **0** | **0** | **0** | **3** | **0** | **3** | **0** | **0** | **0** | **0** | **0** | **3** |
| ***Limnodrilus udekemianus*** | **3** | **0** | **0** | **0** | **0** | **0** | **0** | **2** | **3** | **0** | **3** | **3** | **0** | **0** | **0** | **0** | **0** | **3** |
| ***Branchiura sowerbyi*** | **3** | **0** | **0** | **0** | **0** | **0** | **0** | **2** | **3** | **0** | **3** | **3** | **0** | **0** | **0** | **0** | **0** | **3** |
| ***Helobdella conifer*** | **3** | **0** | **3** | **0** | **0** | **0** | **0** | **0** | **0** | **0** | **3** | **3** | **0** | **0** | **0** | **0** | **3** | **3** |
| ***Batracobdelloides sp.*** | **3** | **0** | **3** | **0** | **0** | **0** | **0** | **0** | **0** | **0** | **3** | **3** | **0** | **0** | **0** | **0** | **3** | **0** |
| ***Limnatis nilotica*** | **3** | **0** | **3** | **0** | **0** | **0** | **0** | **0** | **0** | **0** | **3** | **3** | **0** | **0** | **0** | **0** | **3** | **0** |
| ***Barbronia assiuti*** | **3** | **0** | **3** | **0** | **0** | **0** | **0** | **0** | **0** | **0** | **3** | **3** | **0** | **0** | **0** | **0** | **3** | **0** |
| ***Allolobophora caliginosa*** | **3** | **0** | **0** | **0** | **0** | **0** | **0** | **0** | **0** | **0** | **3** | **0** | **0** | **3** | **0** | **0** | **3** | **0** |
